# TRIM21 attenuates renal carcinoma lipogenesis and malignancy by regulating SREBF1 protein stability

**DOI:** 10.1186/s13046-022-02583-z

**Published:** 2023-01-25

**Authors:** Xintian Chen, Hongmei Yong, Miaolei Chen, Chuyin Deng, Pengfei Wang, Sufang Chu, Minle Li, Pingfu Hou, Junnian Zheng, Zhongwei Li, Jin Bai

**Affiliations:** 1 Cancer Institute, Xuzhou Medical University, 209 Tongshan Road, Jiangsu Province 221004 Xuzhou, China; 2 Center of Clinical Oncology, the Affiliated Hospital of Xuzhou Medical University, Jiangsu Xuzhou, China; 3 Jiangsu Center for the Collaboration and Innovation of Cancer Biotherapy, Cancer Institute, Xuzhou Medical University, 209 Tongshan Road, Jiangsu 221004 Xuzhou, China; 4 Department of Oncology, The Second People’s Hospital of Huai’an, The Affiliated Huai’an Hospital of Xuzhou Medical University, Huaian, Jiangsu China

**Keywords:** TRIM21, Lipogenesis, SREBF1, Ubiquitination, Renal cell carcinoma

## Abstract

**Background:**

Metabolic reprogramming is a hallmark of various cancers. Targeting metabolic processes is a very attractive treatment for cancer. Renal cell carcinoma (RCC) is a type of metabolic disease, and the lipidomic profile of RCC is significantly altered compared with that of healthy tissue. However, the molecular mechanism underlying lipid metabolism regulation in RCC is not clear.

**Methods:**

The XF long-chain fatty acid oxidative stress test kits were used to assess the dependence on long-chain fatty acids and mitochondrial function after knockdown TRIM21 in RCC cells. The effect of TRIM21 on the lipid content in RCC cells was determined by metabolomics analysis, Oil Red O staining, and cellular Nile red staining. qRT-PCR and western blot were used to explore the relationship between TRIM21 and lipogenesis, and then the key molecule sterol regulatory element binding transcription factor 1 (SREBF1) was identified to interact with TRIM21 by immunoprecipitation, which was also identified in an orthotopic model. Subsequently, the relevance and clinical significance of TRIM21 and SREBF1 were analyzed by The Cancer Genome Atlas (TCGA) database, and 239 tissues were collected from RCC patients.

**Results:**

TRIM21 silencing attenuated the dependence of RCC cells on fatty acids, and enhanced lipid accumulation in RCC cells. TRIM21 overexpression significantly decreased lipid contents by decreasing the expression of lipogenic enzymes via ubiquitination-mediated degradation of SREBF1. SREBF1 is critical for TRIM21-mediated lipogenesis inhibition in vitro and in vivo. Moreover, TRIM21 expression is negatively correlated with SREBF1 expression, and TRIM21-SREBF1 is a reliable combinational biomarker for RCC prognosis.

**Conclusion:**

The findings from this study reveal a novel pathway through which TRIM21 inhibits the lipid metabolism process of RCC and shed light on the development of targeted metabolic treatment and prognosis diagnosis of RCC.

**Supplementary Information:**

The online version contains supplementary material available at 10.1186/s13046-022-02583-z.

## Background

Renal cell carcinoma (RCC) is one of the most common malignant tumors of the urinary system, and its incidence is on the rise. More than one-third of RCC patients develop metastasis, and one-fourth experience recurrence [1]. Fully analyzing the molecular mechanism of RCC occurrence and development is of great significance.

Cancer cells usually have a high demand for nutrient metabolism to provide energy and biomass for cellular function and proliferation. Lipids are important substrates for energy metabolism and are also required for cell membrane maintenance and signal transduction. It has been reported that tumor cells with brain metastasis potential have significantly enhanced lipid synthesis characteristics [2]. Currently, ATP-citrate lyase, acetyl-CoA carboxylase, and a variety of drugs, including fat synthase, have entered the clinical trial stage [3], suggesting that lipid anabolic pathways are potential targets for cancer therapy.

TRIM21 belongs to the tripartite motif-containing protein (TRIM) family, and proteins in that family are characterized by a RING domain, B-box domain, coiled-coil domain (CC), and PRY/SPRY region, and have E3 ubiquitin ligase activity [4]. Some key molecules involved in metabolism pathways have also been defined as substrates of TRIM21 [5]. TRIM21 can inhibit glycolysis by ubiquitination and degrading of phosphofructokinase in non-small cell lung cancer [6]. We previously found that the expression of TRIM21 is decreased in RCC tissues and is associated with poor prognosis. TRIM21 inhibits glycolysis in RCC cells by degrading HIF-1α via ubiquitination [7]. The role of TRIM21 in tumor lipid metabolism has also increased in recent years. TRIM21 inhibits fatty acid (FA) synthesis by ubiquitination and degradation of fatty acid synthase (FASN) in hepatocellular carcinoma [8], and further studies found that the ubiquitination of FASN by TRIM21 could be inhibited by glyceronephosphate O-acyltransferase (GNPAT) [9]. Similar to hepatocellular carcinoma, RCC is also accompanied by metabolic reprogramming of lipids [10], and targeting abnormal lipid metabolism is a promising therapeutic target for RCC. However, the detailed mechanism underlying abnormal lipid metabolism has not yet been clarified, and whether TRIM21 is involved in abnormal lipid metabolism in RCC remains unclear.

Sterol regulatory element binding transcription factor 1 (SREBF1) is a core molecule involved in cellular lipid synthesis. Activation of SREBF1 directly triggers the gene transcription of FASN, contributing to the accumulation of lipid droplets and promoting the proliferation of liver cancer cells [11]. Many studies have reported that high expression of SREBF1 is positively associated with poor prognosis in various diseases [12, 13], suggesting that SREBF1 is an attractive therapeutic target. Unfortunately, the regulatory mechanism of SREBF1 has not yet been completely clarified. SREBF1 can be upregulated by SETD8 and E2F1 at the transcriptional level in RCC [14, 15]. Some post-translational modifications also can regulate SREBF1 expression. Small ubiquitin-related modifier (SUMO) E3 ligase can sumoylate SREBF1 at Lys98, leading to suppression of the hepatic lipogenesis in response to fast-induced signals [16]. SREBF1 is a substrate for neddylation by the NEDD8-conjugating enzyme UBC12, and neddylation stabilizes SREBF1 with decreased ubiquitylation, contributing to the aggressive malignant phenotype of HCC and breast cancer [17]. At present, the most important E3 ubiquitin ligase of SREBF1 is SCF^Fbw7^ [18], and phosphorylation of SREBF1 at Thr426, Ser430 and Ser434 inhibits the ubiquitination of SCF^Fbw7^ [18–20]. However, whether there are other E3 ubiquitin ligases in SREBF1 remains unclear. Identifying a new E3 ubiquitin ligase of SREBF1 is of great potential and significance for the development of drugs targeting SREBF1, balancing the lipid metabolism process, and providing new ideas for the treatment of tumors.

Here, we discovered that TRIM21 inhibits lipid accumulation in RCC cells and prevents renal carcinoma tumorigenesis. Mechanistically, TRIM21 is a novel E3 ligase of SREBF1, and the SPRY domain of TRIM21 can bind to SREBF1 and mediate its ubiquitination degradation in a K63-linked manner. The degradation of SREBF1 leads to the decreased transcriptional expression of downstream lipid metabolism-related genes and therefore fine-tunes the lipid metabolism process of RCC cells. Moreover, SREBF1 expression is inversely correlated with TRIM21 expression in clinical tissue samples, and SREBF1 and TRIM21 are reliable combinational biomarkers for RCC prognosis. Overall, findings from our study reveal a novel pathway through which TRIM21 inhibits the lipid metabolism process of RCC cells and sheds light on the development of targeted metabolic treatment of RCC.

## Materials and methods

### Patients and sample collection

Tissue microarray (TMA) including 239 RCC tissues were enrolled at the Affiliated Hospital of Xuzhou Medical University from 2005 to 2008 in China. Patients’ clinicopathologic information was obtained from the medical records of the Affiliated Hospital of Xuzhou Medical University. Cancerous and para-cancerous tissues from six RCC patients were also collected from 2021 to 2022.

### Cell culture and treatment

The RCC cell lines were obtained from the cell bank of the Chinese Academy of Sciences. ACHN, 786-O, and Caki-1 cells were cultured in DMEM supplemented with 10% fetal bovine serum, 100 U/ml penicillin, and 100 μg/ml streptomycin and incubated at 37 °C in a humidified incubator with 5% CO_2_.

Small interfering RNAs (siRNAs, 50 nM) against human TRIM21 and SREBF1 were purchased from GenePharma Technology (Shanghai, China), and were performed as previously described [21], the sequences of negative control and siRNAs are available in the Supplementary Materials and Methods. The overexpression plasmid (Flag-SREBF1) and knockdown plasmid (sh-SREBF1 and sh-TRIM21) were purchased from Miaolingbio (Wuhan, China), and were transfected into RCC cells with Lipofectamine 2000 (Invitrogen, Shanghai, China) as previously described [22].

### Western blot analysis and antibodies

Western blot analysis was performed as previously described [23, 24]. Specific primary antibodies against GAPDH (60004–1-Ig, Proteintech), TRIM21 (12108–1-AP, Proteintech), SREBF1 (14088–1-AP, Proteintech), FASN (10624–2-AP, Proteintech), GPAM (A6610, ABclonal), ATGL (55190–1-AP, Proteintech), Perilipin-1 (DF-7602, Affinity Biosciences), MGLL (14986–1-AP, Proteintech), ACLY (15421–1-AP, Proteintech), HSL (17333–1-AP, Proteintech) were used for Western blot assays.

### RNA extract, reverse transcription-PCR, and qRT-PCR

These relevant protocols were carried out as previously described [25]. The detailed method and primers used for quantitative qRT-PCR analysis are listed in the Supplementary Materials and Methods.

### Immunohistochemistry (IHC) staining

IHC assays were performed following a standard streptavidin-peroxidase method as previously reported [26]. For primary antibody, Anti-TRIM21 antibody was used with 1:400 dilution, Anti-SREBF1 was used with 1:500 dilution, Anti-FASN was used with 1:500 dilution, Anti-MGLL was used with 1:200 dilution, Anti-HSL was used with 1:500 dilution, Anti-Ki-67antibody (12202S, CST) was used with 1:200 dilution. The detailed method of IHC assessment was described in the Supplementary Materials and Methods.

### Seahorse assays

The XF long-chain fatty acid oxidative stress test kit was used to access the dependence on long-chain fatty acids and mitochondrial function using Seahorse Bioscience Extracellular Flux Analyzer (XF96, Seahorse Bioscience Inc., North Billerica, MA, USA) by measuring oxygen consumption rate (OCAR) in real-time as described in manufacturer’s instructions. The detailed procedure was described in the Supplementary Materials and Methods.

### Metabolomics and data analysis

ACHN and ACHN stably overexpressed TRIM21 cells were cultured in six repeats. The cells were harvested using trypsin and washed three times with cold PBS, and the cell pellets were shipped to Shanghai Applied Protein Technology Co. Ltd for metabolomics and data analysis.

### Cellular Nile red staining

Forty eight hours after transfection, the renal cell lines (ACHN, 786-O, Caki-1) were counted and plated into a six-well plate (1 × 10^5^/ cell per well). 24 hours later, the cells were stained with 1 μm Nile Red (KGDYE22190; KeyGen Biotech, Jiangsu, China) and DAPI (VIC112; VICMED, Xuzhou, China) for 10 min at room temperature. Images were obtained with a fluorescence microscope. As for flow cytometry, the cells were only stained with Nile Red for 10 min, and the fluorescence intensity of each group was detected.

### Oil Red O staining

Forty eight hours after transfection, the renal cell lines (ACHN, 786-O, Caki-1) were counted and plated into a six-well plate (1 × 10^5^/ cell per well). 24 hours later, Oil Red O (G1260, Solarbio Life Sciences, Beijing, China) staining was performed as manufacturer’s instructions. The detailed procedure was described in the Supplementary Materials and Methods.

### Stable cell line generation

Stable cells were generated using lentivirus through a previously described method [27]. The detailed procedure was described in the Supplementary Materials and Methods.

### Animal work

NSG mice (6–8 weeks old) were purchased from SHANGHAI MODEL ORGANISMS (Shanghai, China). All animal experiments were approved by the Animal Care and Use Committee at Xuzhou Medical University. The stable cells were resuspended in a serum-free medium (5 × 10^7^ cells /mL). NSG mice were randomly divided into the control group (Vector), TRIM21 overexpression group (OE-TRIM21), SREBF1 overexpression group (OE-SREBF1), and TRIM21 + SREBF1 double overexpression group (OE-TRIM21 + OE-SREBF1). Mice were anesthetized with isoflurane, after the kidney was exposed from the posterior side, the cell suspension (5 × 10^5^ cells /10μL) was injected under the renal capsule, and the wound was sutured and sterilized. Every 7 days in vivo imaging of small animals was used to monitor tumor size and metastasis. The experiment was terminated when the difference in tumor growth between groups was fully developed. The orthotopic tumors were collected for frozen sections to detect the formation of lipid droplets. Paraffin embedding and histochemical staining were performed to detect the expression of lipid metabolism-related indicators.

### Database analysis relevance and clinical significance of TRIM21 and SREBF1

RNA-sequencing expression (level 3) profiles and corresponding clinical information for RCC were downloaded from The Cancer Genome Atlas (TCGA) database (https://portal.gdc.com). The detailed analysis was described in the Supplementary Materials and Methods.

### Statistical analysis

Statistical analyses were conducted using SPSS 20.0 software (SPSS Inc., Chicago, IL, USA) and GraphPad Prism 8. The association between TRIM21 and SREBF1 expression and the clinicopathologic parameters of patients with RCC was evaluated by Chi-square test. Three sets of data from the single-factor analysis were evaluated using one-way ANOVA corrected with Tukey’s multiple comparison test (single-factor comparison). Statistical differences between control and overexpression groups were analyzed by two-tailed Student’s t-test. Data were presented as the mean squared error (SEM), and the bar indicates the mean. *p* < 0.05 was considered statistically significant.

## Results

### TRIM21 silencing attenuates the dependence of RCC cells on fatty acids

Metabolic reprogramming is essential in maintaining homeostasis, and various central metabolism pathways involved in glucose and fatty acids can be dysregulated in cancer cells [28]. Compared with normal tissues, cancer cells have a higher demand for fatty acids to generate lipid membranes and precursors for signaling molecules [29]. TRIM21 acts at multiple nodes to control cancer metabolic reprogramming by inhibiting the increased metabolic demand of malignancy. Our previous study found that TRIM21 enhances the glycolytic capacity of RCC cells. Whether TRIM21 participates in metabolic reprogramming of lipids in RCC remains unknown.

**Fig. 1:**
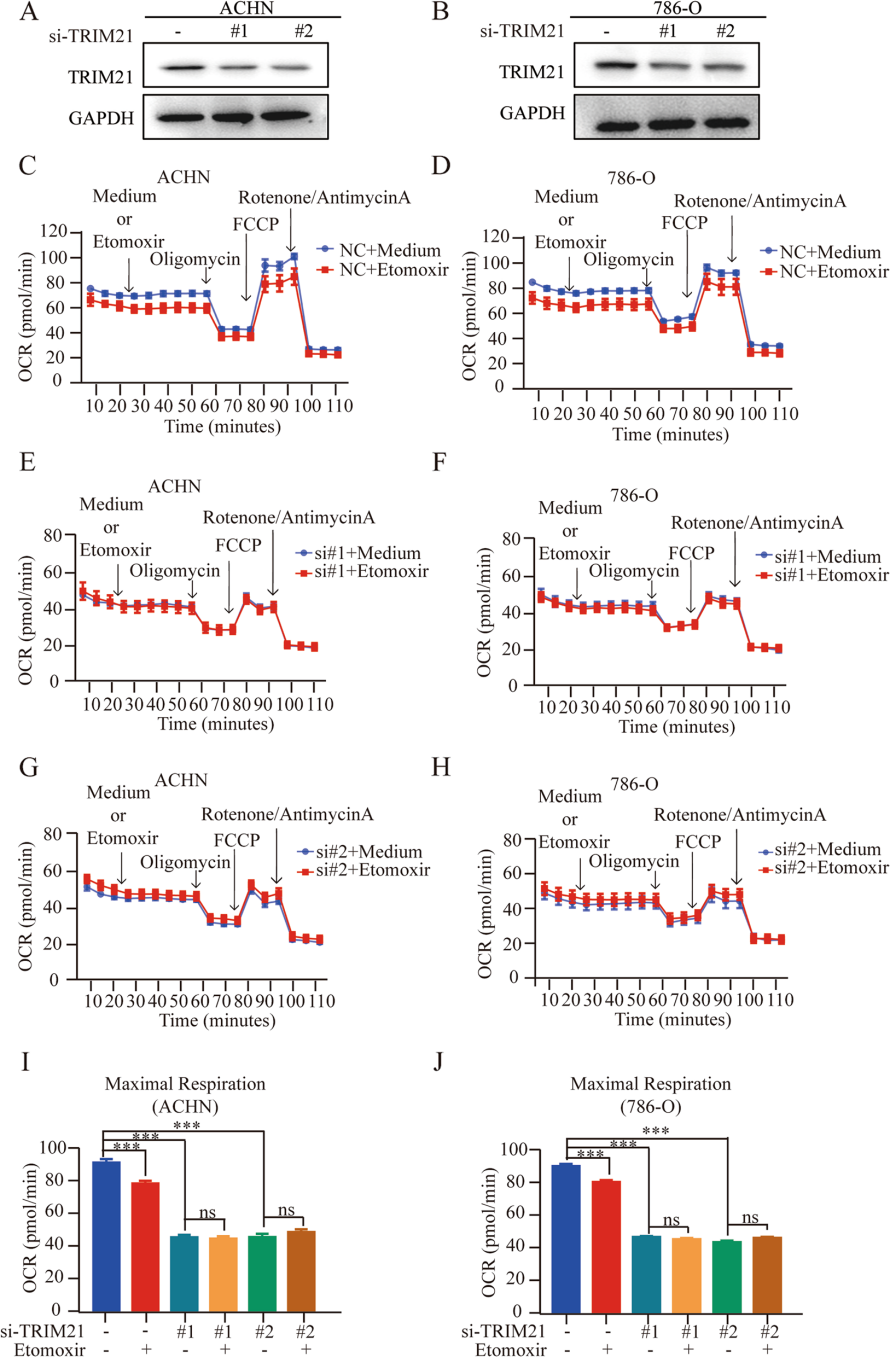
TRIM21 silencing attenuates the dependence of RCC cells on fatty acids. **A** and **B** Western blot was used to detect the efficiency of TRIM21 knockdown in ACHN and 786-O, and GAPDH was used as a loading control. **C**-**H** Oxygen consumption rate (OCAR) was analyzed when TRIM21 was knockdown in ACHN and 786-O by Seahorse analysis. **I**-**J** The maximum respiratory capacity was analyzed when TRIM21 was knockdown in ACHN and 786-O. All the results were confirmed by three times repeated experiments. Statistical analysis was performed using unpaired t-tests. All statistical tests were two-sided. *p < 0.05, **p < 0.01, ***p < 0.001

Considering the abnormal accumulation of lipids in RCC, we first used the XF long-chain fatty acid oxidative stress test kit to assess the dependence on FAs when TRIM21 was knocked down in RCC cells (Fig. 1A-B). Etomoxir inhibits FA oxidation by inhibiting carnitine palmitoyl transferase 1A (CPT1a) [30]. The results showed that after Etomoxir treatment, the oxygen consumption rate (OCAR) in ACHN and 786-O cells (Fig. 1C-D) was decreased, and the maximum respiratory capacity was also weakened (Fig. 1I-J), which means that the metabolic process of RCC cells depends on fatty acids. However, when TRIM21 was knocked down in ACHN and 786-O cells, the etomoxir treatment had little effect on the maximum respiratory capacity (Fig. 1E-J). These results suggested that TRIM21 deficiency attenuates the dependence of RCC cells on FAs, which leaves us with a question to ponder: since FAs are not consumed by mitochondria, where do they go?

### TRIM21 silencing enhances lipid accumulation in RCC cells

Lipid droplets (LDs) are generally conserved dynamic organelles that can store and mobilize FAs and other lipid species; they increase the accumulation of lipid reserves to meet the energy needs of the cells while avoiding cytotoxicity and promoting its malignant progression [31].

**Fig. 2:**
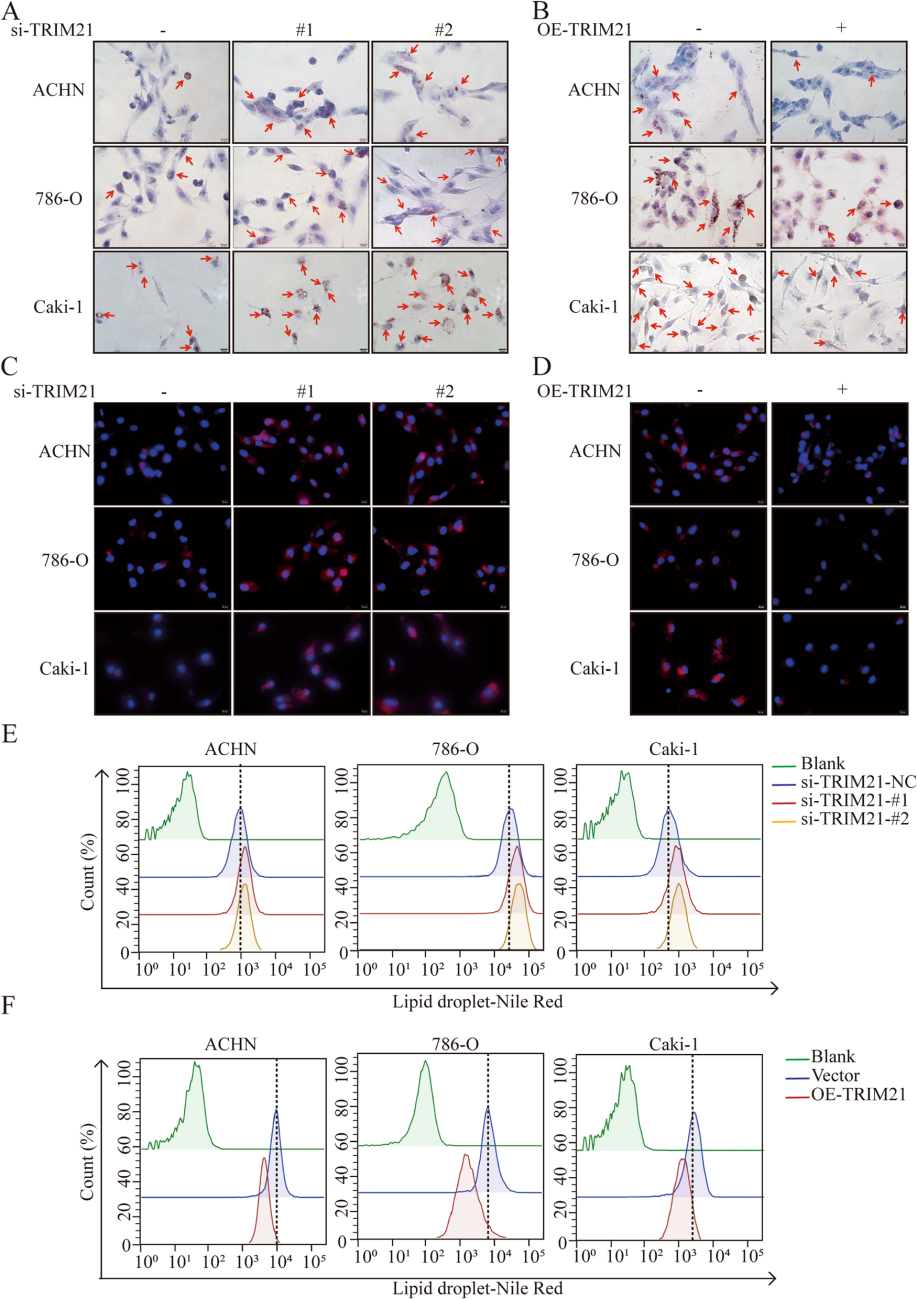
TRIM21 silencing enhances lipid accumulation in RCC cells. **A**-**B** Representative images of Oil Red O staining when TRIM21 was knocked down or overexpressed in ACHN, 786-O, and Caki-1 cells (red arrows indicate lipid droplets). **C** Nile red staining and fluorescence microscopy were performed when TRIM21 was knocked down in ACHN, 786-O, and Caki-1 cells. The red fluorescence intensity of labeled lipid droplets increased (red fluorescence indicates lipid droplets, blue fluorescence DAPI staining nuclear). **D** Nile red staining and fluorescence microscopy were performed when TRIM21 was over-expressed in ACHN, 786-O, and Caki-1 cells. The red fluorescence intensity of labeled lipid droplets decreased (red fluorescence indicates lipid droplets, blue fluorescence DAPI staining nuclear). **E** Flow cytometry was used to access the fluorescence intensity of Nile red staining. The fluorescence intensity of labeled lipid droplets was enhanced when TRIM21 was knocked down. **F** The fluorescence intensity of labeled lipid droplets was decreased when TRIM21 was over-expressed. All the results were confirmed by three times repeated experiments

RCC, especially clear cell renal cell carcinoma (CCRCC), is characterized by large intracellular LDs [32]. We hypothesized that TRIM21 deficiency might cause unutilized FAs to be stored in LDs in RCC cells. Therefore, Oil Red O staining was used to further investigate the content of LDs in ACHN, 786-O, and Caki-1 cells. When TRIM21 was knocked down, the LDs content was increased significantly (Fig. 2A), while TRIM21 overexpression decreased the content (Fig. 2B).

Nile red staining, which is also an indicator of LDs content, was performed, and fluorescence microscopy and flow cytometry were used to confirm the results. Similar to Oil Red O staining, TRIM21 knockdown cells had a strong Nile red staining intensity (Fig. 2C), and TRIM21 overexpression led to a weak intensity (Fig. 2D). Flow cytometry showed similar results (Fig. 2E-F). Overall, the above results strongly suggest that TRIM21 deficiency increased lipid droplet contents in RCC cells.

### TRIM21 overexpression significantly decreases lipid contents in RCC cells

**Fig. 3:**
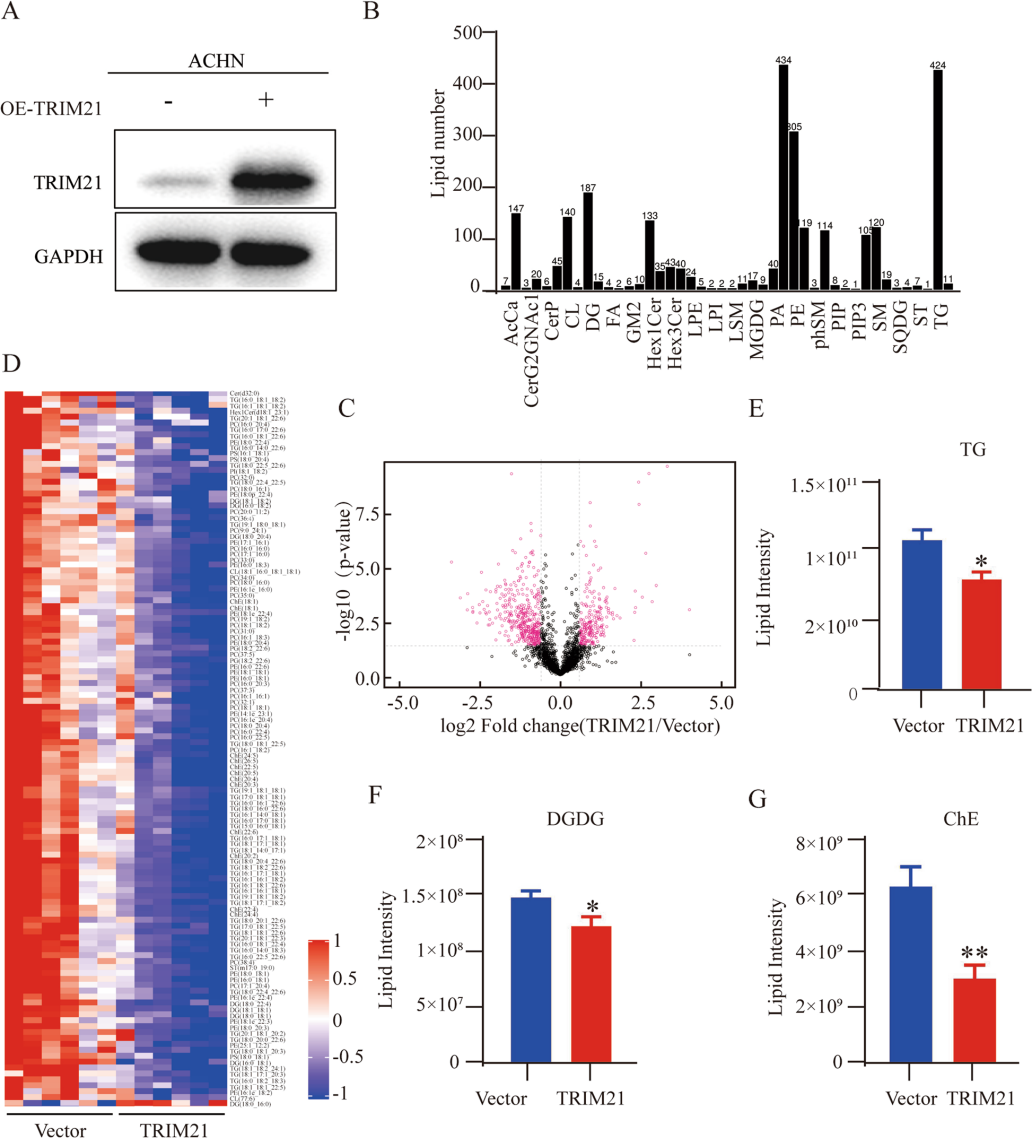
TRIM21 overexpression significantly decreases lipid contents in RCC cells. **A** Western blot was used to detect the stable overexpression efficiency of TRIM21 in ACHN. GAPDH was used as a loading control. **B** The number of lipid subclasses and lipid molecules was detected by mass spectrometry. **C** The volcano plot shows the overall differential expression of lipid molecules. The rose-red dots show the differential lipid molecules screened by univariate statistical analysis. **D** The hierarchical clustering results of different lipids in the control group (Vector) and TRIM21 overexpression group (TRIM21). The horizontal coordinate represents the group of samples, and the vertical coordinate represents the different lipid molecules. The results showed that the expression levels of various lipid molecules in the TRIM21 group were significantly lower than those in the Vector group. The relative contents of (**E**) triglyceride (**F**) diglycerol and (**G**) cholesterol in the OE-TRIM21 group were significantly lower than those in the Vector group. *p < 0.05, **p < 0.01

Lipid metabolism is complex, and lipids and lipid-derived products are large and diverse (e.g., diacylglycerol, cholesterol derivatives, and phosphatidic acid) [33]. To further clarify the biological phenomenon of attenuation of lipid content by TRIM21, untargeted lipidomic analysis was performed in ACHN (Vector) and ACHN stably overexpressing TRIM21 (OE-TRIM21) cells (Fig. 3A).

The number of lipid classes and lipid species was identified (Fig. 3B), and the overall differential expression of lipid molecules in the two groups was clearly illustrated by the volcano map (Fig. 3C). To explore the different contents of lipids in the two groups more comprehensively and visually, hierarchical clustering was conducted to accurately screen the marker lipids and study the changes in related metabolic processes (Fig. 3D). The results showed that the OE-TRIM21 group had lower contents of phosphatidylcholines (PC), phosphatidyl ethanolamine (PE), triacylglycerols (TG), diacylglycerols (DG), and cholesterol (ChE). The relative content of lipid subclasses in the Vector and OE-TRIM21 groups was analyzed further, and we found that compared with other lipid subclasses, the content of TG, DG, and ChE in OE-TRIM21 cells decreased significantly (Fig. 3E-G). These results further verified the ability of TRIM21 to reduce lipid accumulation in RCC cells.

### TRIM21 decreases the expression of lipogenic enzymes by mediating ubiquitination-mediated degradation of SREBF1

Tumor cells can undergo metabolic reprogramming by regulating the expression of metabolic enzymes to satisfy cell biosynthesis and energy requirements. The primary neutral lipids of the LD core are triacylglycerol and steryl esters, which are coated by a phospholipid monolayer that solubilizes the LDs in the cytoplasm. Neutral lipid metabolism depends on the precisely coordinated mechanisms of various enzymes to maintain cellular homeostasis [34].

We further explored changes in the mRNA and protein levels of some key metabolic enzymes involved in lipid synthesis and decomposition when TRIM21 was knocked down or over-expressed. The lipid metabolic enzymes and metabolic-associated key genes we examined included SREBF1, ATP citrate lyase (ACLY), FASN, and stearoyl-CoA desaturase 1 (SCD1) [35], which participate in FA metabolism; glycerol-3-phosphate acyltransferase mitochondrial (GPAM), patatin-like phospholipase domain containing 2 (ATGL), monoglyceride lipase (MGLL), hormone sensitive lipase (HSL) and perilipin-1, which are involved in triglyceride metabolism [36–38]; ceramide synthase 6 (CERS6) and Lipin-1, which are involved in phospholipid metabolism [39, 40].

**Fig. 4:**
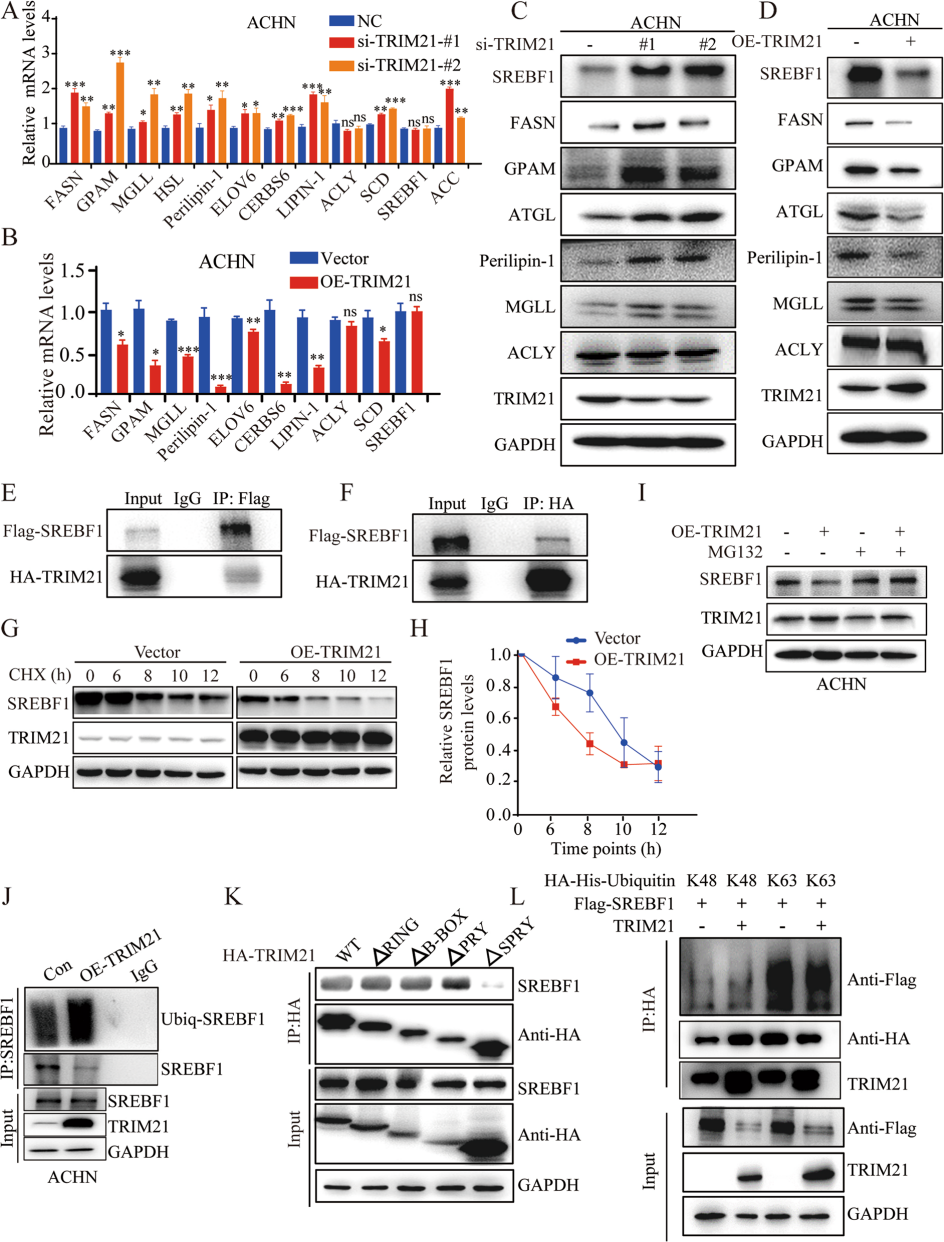
TRIM21 decreases the expression of lipogenic enzymes by mediating ubiquitination-mediated degradation of SREBF1. TRIM21 knockdown (**A**) and overexpression in ACHN (**B**) can increase and decrease metabolic enzymes above the transcriptional level, with the exception of ACLY and SREBF. TRIM21 knockdown (**C**) and overexpression in ACHN (**D**) can increase and decrease metabolic enzymes above at the protein level except ACLY. ACHN was transfected with exogenous HA-TRIM21 and Flag-SREBF1. Anti-FLAG (**E**) and anti-HA (**F**) antibodies were used for immunoprecipitation to detect the mutual binding between TRIM21 and SREBF1. **G-H** Western blot showing SREBF1 protein decay at the indicated time points after cycloheximide (50 μg/ml) addition to ACHN. **I** Western blot showing the effects of the proteasome inhibitor MG132 (10 μM for 6 h) treatment on SREBF1 protein accumulation. **J** TRIM21 was overexpressed in ACHN cells, and immunoprecipitation was performed using an Anti-SREBF1 antibody, which suggested that TRIM21 could bind to SREBF1 and increase the ubiquitination degradation level of SREBF1. **K** HEK293T cells were transfected with TRIM21 WT or the indicated mutant for 48 h harvested and subjected to IP with anti-HA beads. **L** K48-only and K63-only ubiquitin-HA plasmids alone or co-transfected with TRIM21 and Flag-SREBF1 plasmid into HEK293T cells for 48 h. Then, the cells were harvested and subjected to IP with anti-Flag beads. All the results were confirmed by three times repeated experiments. Statistical analysis was performed using unpaired t-tests. All statistical tests were two-sided. *p < 0.05, **p < 0.01, ***p < 0.001, ns, no significance

The qRT-PCR results showed that TRIM21 knockdown upregulated the expression of most of these metabolic enzymes at the transcriptional level, whereas TRIM21 overexpression downregulated the mRNA expression of the above-mentioned genes. Interestingly, the mRNA levels of SREBF1 and ACLY were not altered by TRIM21 knockdown or ectopic TRIM21 expression in RCC cells (Fig. 4A-B and Supplementary Fig. 1A-B). We further examined the expression of these lipid metabolism genes at the protein level. The results showed that TRIM21 knockdown or overexpression did not affect the expression of ACLY. However, at the protein level, TRIM21 deficiency increased the expression of genes related to lipid metabolism, including SREBF1, FASN, GPAM, ATGL, perilipin-1, and MGLL (Fig. 4C and Supplementary Fig. 1C), and overexpression of TRIM21 decreased the expression of those genes (Fig. 4D and Supplementary Fig. 1D).

SREBF1 primarily regulates genes involved in the synthesis of fatty acids, triglycerides, and phospholipids [41]. CERS6 and Lipin-1 are transcriptionally activated by SREBF1 [39, 40]. Considering the different regulatory effects of TRIM21 on the expression of SREBF1 at the protein and mRNA levels (i.e., TRIM21 only regulates SREBF1 expression at the protein level), we speculate that TRIM21 may attenuate SREBF1 expression by regulating its protein stability and that TRIM21 may restrain lipogenesis in an SREBF1–dependent manner.

To confirm our hypothesis, HA-TRIM21 and FLAG-SREBF1 plasmids were transferred into ACHN cells simultaneously, and immunoprecipitation was performed to detect whether there was an interaction between TRIM21 and SREBF1. The results showed that TRIM21 and SREBF1 can indeed bind to each other (Fig. 4E-F).

Next, we detected SREBF1 protein expression in RCC cells after treatment with cycloheximide (CHX) or the proteasome inhibitor MG132. The data indicated that CHX shortened the half-life of SREBF1 (Fig. 4G-H), and MG132 inhibited the ubiquitination degradation of SREBF1 (Fig. 4I) in ACHN cells when TRIM21 was over-expressed. TRIM21 overexpression led to increased ubiquitination degradation of SREBF1 in ACHN and 786-O cells (Fig. 4J and Supplementary Fig. 1E) by immunoprecipitation analysis.

To further explore the specific domain of TRIM21 that binds to SREBF1, truncated mutants of TRIM21 were used [42]. The results suggested that truncated mutants without their SPRY domains had impaired interactions with SREBF1 (Fig. 4K), indicating that the SPRY domain is mainly required for the interaction of TRIM21 with SREBF1. IP assays further confirmed that the ubiquitination of SREBF1 induced by TRIM21 occurred mainly via the K63 linkage (Fig. 4L). The results demonstrate that TRIM21 has a general regulatory effect on SREBF1 ubiquitination in RCC cells. The SPRY domain of TRIM21 mainly interacts with SREBF1, and TRIM21 delivers K63-ubiquitin to SREBF1, leading to the recognition and degradation of SREBF1 by the proteasomes, which may further attenuate the lipogenesis of RCC cells.

### SREBF1 is critical for TRIM21-mediated lipogenesis inhibition in vitro

Our results show that TRIM21 decreases SREBF1 protein stability by elevating its ubiquitination degradation. We propose that the inhibition of lipid accumulation mediated by TRIM21 may depend on a decrease in SREBF1 in RCC cells.

**Fig. 5:**
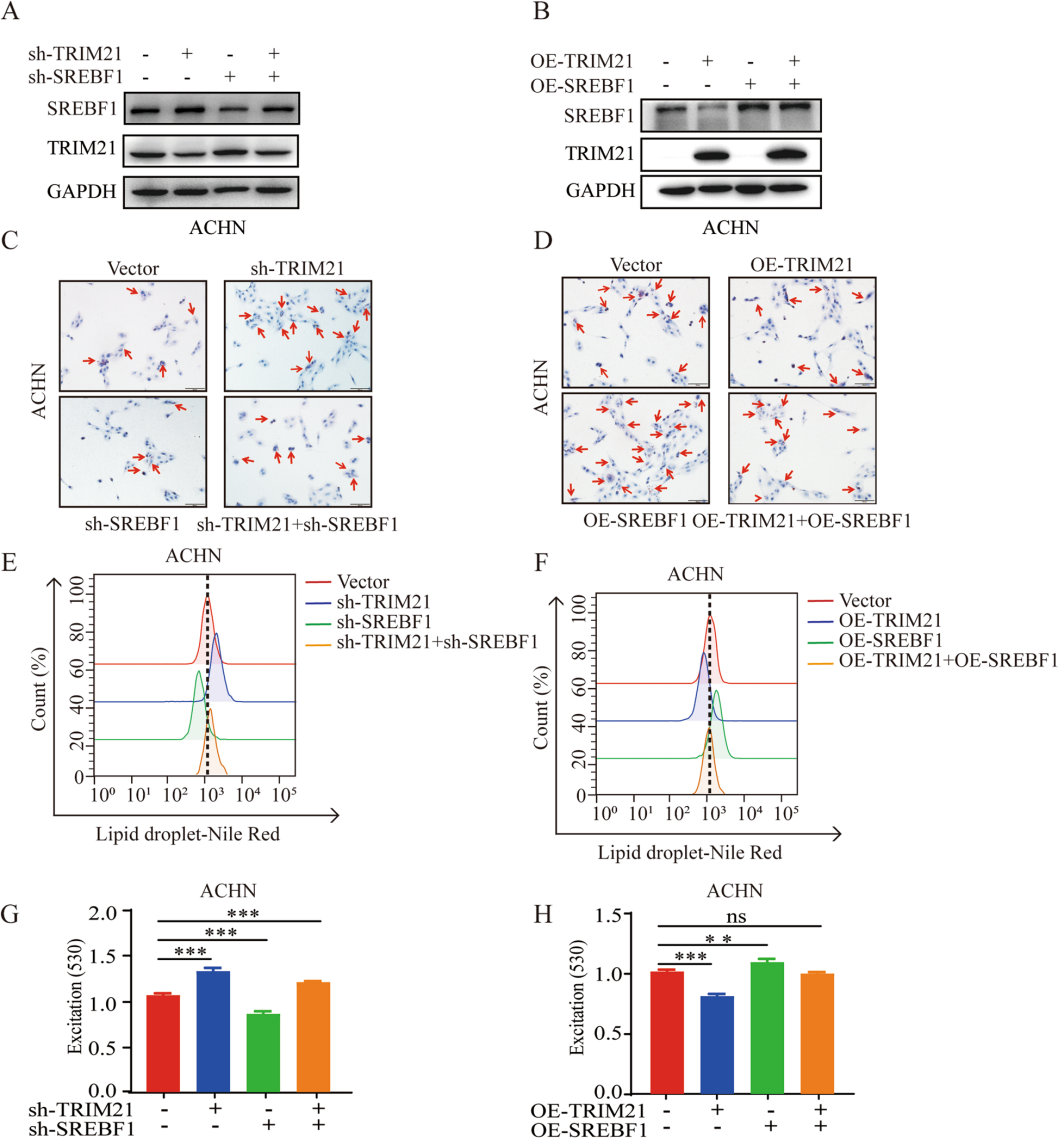
SREBF1 is critical for TRIM21-mediated lipogenesis inhibition in vitro. **A** and **B** Western blot was used to detect the stable knockdown and overexpressed efficiency of TRIM21 and SREBF1 alone or combination with in ACHN, and GAPDH was used as a loading control. Representative images of Oil Red O staining when TRIM21 and SREBF1 were stably knocked-down (**C**) or over-expressed (**D**) alone or combination with in ACHN cells (red arrows indicate lipid droplets). Flow cytometry (**E**) and a microplate reader (**G**) were used to access the fluorescence intensity of Nile red staining when TRIM21 and SREBF1 were stably knocked down alone or simultaneously. Flow cytometry (**F**) and a microplate reader (**H**) were used to access the fluorescence intensity of Nile red staining when TRIM21 and SREBF1 were stably overexpressed alone or simultaneously. All the results were confirmed by three times repeated experiments. Data are presented as the means ± SEM for experiments in triplicate. ns, no significance, **p < 0.01, ***p < 0.001

Flow cytometry fluorescence intensity and absorbance measured by a microplate reader were used to assess the content of lipid droplets in ACHN and 786-O when SREBF1 and TRIM21 were knocked down or overexpressed simultaneously (Fig. 5A-B, Supplement Fig. 2A, B and G). Compared to the control group, TRIM21 knockdown enhanced Oil Red O staining, fluorescence intensity representing lipid droplet content; and lipid droplet content was weakened when SREBF1 was knocked down. As expected, when TRIM21 and SREBF1 were knocked down simultaneously, the lipid droplet content was similar to that in the vector group (Fig. 5C, E, G, and Supplement Fig. 2C-F). These results indicate that SREBF1 knockdown reversed the increase in lipid droplet content caused by TRIM21 knockdown. Moreover, SREBF1 expression led to increased Oil Red O staining and fluorescence intensity as expected, and the reduced lipid droplet content induced by TRIM21 overexpression was also reversed by SREBF1 overexpression (Fig. 5D, F, H, and Supplement Fig. 2H-L). These results suggest that the regulatory effect of TRIM21 on lipogenesis in RCC cells is mainly dependent on SREBF1.

### TRIM21 inhibits RCC tumorigenesis by modulating SREBF1-regulated lipid accumulation in an orthotopic model

Most of our above studies have demonstrated an inhibitory effect of TRIM21 on lipid accumulation at the cellular level in vitro. Moreover, we explored whether TRIM21 suppresses lipid accumulation in RCC cells in vivo. A renal orthotopic tumor model was established to further evaluate whether TRIM21 can decrease lipid droplet content in vivo.

**Fig. 6:**
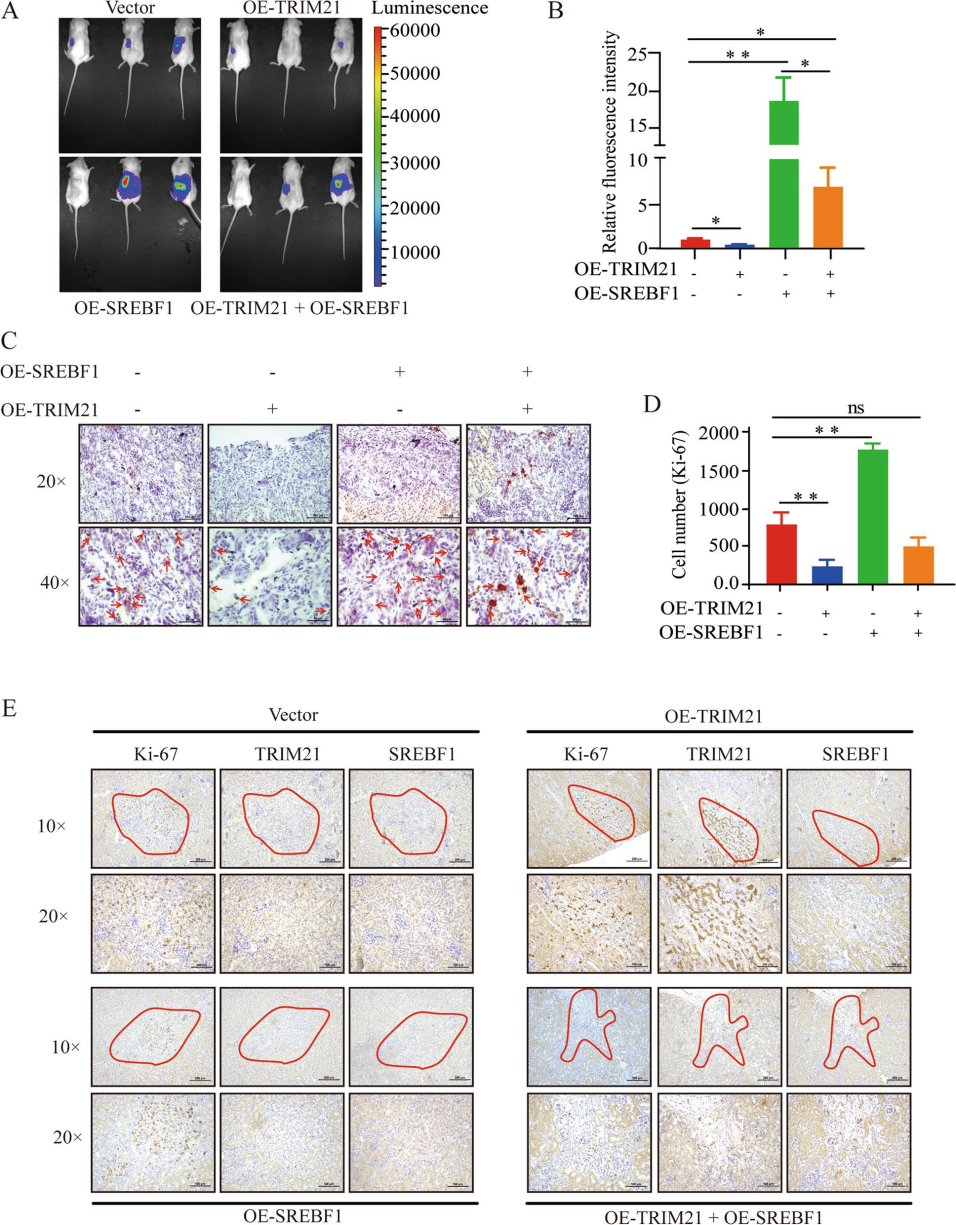
TRIM21 inhibits RCC tumorigenesis by modulating SREBF1-regulated lipid accumulation in an orthotopic model. NSG mice were randomly divided into a control group (Vector), TRIM21 overexpression group (OE-TRIM21), SREBF1 overexpression group (OE-SREBF1), and TRIM21 + SREBF1 double overexpression group (OE-TRIM21 + OE-SREBF1). The cell suspension (5 × 10^5^cells/10μL) was injected under the renal capsule. Representative in vivo imaging images of each group was shown in (**A**), and the fluorescence intensity of each group was counted in **B**. **C** Representative images of Oil Red O staining of renal orthotopic tumor in each group. **D-E** IHC detected Ki-67 expression and quantification of Ki-67-positive cells in tumors. **E** Representative examples of IHC-based correlation between TRIM21 and SREBF1 expression in the renal orthotopic tumor. Statistical analysis was performed using unpaired t-tests. All statistical tests were two-sided. **p < 0.01, ***p < 0.001

NSG mice were divided into four groups and inoculated with luciferase-labeled ACHN cells (Vector), stably overexpressing TRIM21-ACHN cells (OE-TRIM21), stably overexpressing SREBF1-ACHN cells (OE-SREBF1) and simultaneously stably overexpressing TRIM21 and SREBF1-ACHN cells (OE-TRIM21 + OE-SREBF1) in the kidney in situ, and fluorescence expression was detected. As shown in Fig. 6A-B, compared with the Vector group, the OE-TRIM21 group had a lower fluorescence intensity; the OE-TRIM21 + OE-SREBF1 group had a lower fluorescence intensity than the OE-SREBF1 group, and both of those groups had higher fluorescence intensities than that of the Vector group.

The tumors in each group were cryosectioned and stained with Oil Red O to detect lipogenesis. The results were consistent with the in vitro data: compared with the Vector group, the Oil Red O staining was reduced in the OE-TRIM21 group, enhanced in the OE-SREBF1 group, and the OE-TRIM21 + OE-SREBF1 group had a similar Oil Red staining (Fig. 6C). Ki-67 expression was used to define the location of the tumor in situ and the proliferative capacity of the cells. The IHC results showed that the OE-SREBF1 group had more positive cells, and the OE-TRIM21 + OE-SREBF1 group had Ki-67-positive cells, similar to the Vector group (Fig. 6D-E). Consistent with the in vitro results, high TRIM21 expression was associated with low SREBF1 expression in the OE-TRIM21 group (Fig. 6E). These results further demonstrated that TRIM21 can suppress lipogenesis by regulating SREBF1 expression in vivo.

### TRIM21 is negatively correlated with SREBF1 expression, and TRIM21-SREBF1 is a reliable combinational biomarker for RCC prognosis

Our data strongly suggest that TRIM21 can inhibit lipid metabolism in RCC cells by mediating SREBF1 degradation. Accordingly, we wanted to explore the relevance and clinical significance of TRIM21 and SREBF1 in RCC patients.

**Fig. 7:**
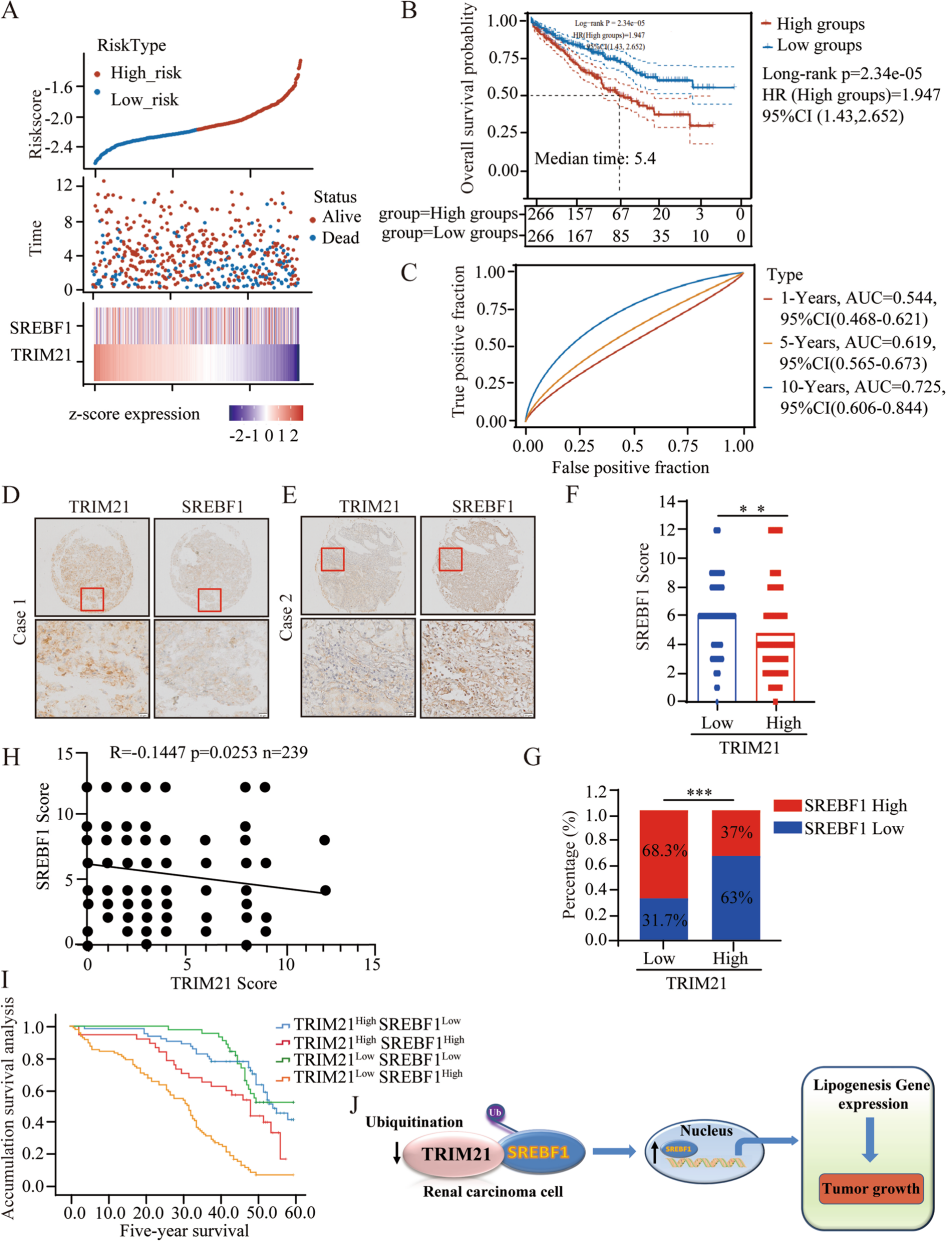
TRIM21 is negatively correlated with SREBF1 in RCC patients and TRIM21-SREBF1 is a reliable combinational biomarker for RCC prognosis. **A** The Riskscore, survival time, and survival status of the RCC dataset. The top scatterplot represents the Riskscore from low to high. Different colors represent different groups. The scatter plot distribution represents the Riskscore of different samples corresponding to the survival time and survival status. The bottom heatmap is the SREBF1 and TRIM21 expression from the signature. **B** Kaplan–Meier survival analysis of the risk model from the RCC dataset, comparison among different groups was made by log-rank test. HR (High groups) represents the hazard ratio of the low-expression sample relatives to the high-expression sample. HR > 1 indicates that TRIM21 and SREBF1 are combinational risk factors. HR (95%Cl), the median survival time (LT50) for different groups, in years. **C** The ROC curve and AUC of the TRIM21 and SREBF1. The higher values of AUC correspond to higher predictive power. **D-E** Representative immunohistochemistry images of TRIM21 and SREBF1 protein expression in carcinoma tissues in RCC patients. **F-G** Analyzing the relevant SREBF1 expression in TRIM21-low cases and TRIM21-high cases. **H** The correlation between TRIM21 and SREBF1 expression was analyzed in RCC tissues according to the scores of immunohistochemistry staining by Pearson’s correlation coefficient test. **I** Overall survival rates were analyzed between four subgroups (TRIM21^high^/SREBF1^low^, TRIM21^high^/SREBF1^high^, TRIM21^low^/SREBF1^low^, TRIM21^low^/SREBF1^high^). **J** A cartoon summarizing our findings. TRIM21 binds to SREBF1, a transcription factor that has a central role in lipogenesis, and mediates the ubiquitination degradation of SREBF1, leading to the decreased transcriptional expression of downstream lipid metabolism-related genes, and therefore fine-tunes the lipid metabolism process of RCC. Statistical analysis was performed using unpaired t-tests. All statistical tests were two-sided. **p < 0.01, ***p < 0.001

Analysis results from The Cancer Genome Atlas (TCGA) database showed that low TRIM21 expression was accompanied by high SREBF1 expression, which represents the high-risk group (Fig. 7A). Notably, low TRIM21 expression was not accompanied by significant SREBF1 expression, which may be caused by other regulatory mechanisms of SREBF1 at the transcriptional level. The Kaplan–Meier survival analysis also showed that the high-risk group had a shorter survival period (Fig. 7B), and the AUC for 10-year overall survival (OS) was 0.725, which corresponded to a higher predictive power (Fig. 7C). These results further suggest that TRIM21 and SREBF1 may be reliable combinational biomarkers for RCC prognosis.

Moreover, histochemical staining of TRIM21 and lipid metabolism-related markers in carcinoma and adjacent tissues from six RCC patients was performed to preliminarily explore the potential relevance of TRIM21 and SREBF1. These results indicated that compared with adjacent tissues, the expression of TRIM21 in cancer tissues was lower, whereas SREBF1 and other markers related to lipid metabolism were expressed at higher levels expression in tumor tissues (Supplementary Fig. 3). These results suggest that the expression of markers related to lipid metabolism may be negatively correlated with TRIM21 expression in RCC tissues.

We detected TRIM21 and SREBF1 expression levels in 239 RCC samples and examined their relevance and clinical significance further. Fisher’s exact tests were used to examine the correlation of TRIM21 and SREBF1 expression between clinicopathological characteristics. TRIM21 expression was low in 58.2% (139/239) and high in 41.8% (100/239) of the RCC tissues. Low TRIM21 expression was significantly positively correlated with tumor size (*p* = 0.001), lymph node metastasis (*p* = 0.004), and distant metastasis (*p* = 0.001) (Supplementary Table 1). However, SREBF1 expression was high in 55.2% (132/239) and low in 44.8% (107/239) of the RCC tissues. High SREBF1 expression was significantly positively correlated with tumor size (*p* = 0.005), lymph node metastasis (*p* = 0.003), and distant metastasis (*p* = 0.000) (Supplementary Table 2). Univariate and multivariate Cox regression models were used to further investigate the role of TRIM21 and SREBF1 in patients with RCC (Supplementary Table 3, Tables 4, and Table 5). Our results confirmed that TRIM21 and SREFB1 expression may serve as potential independent prognostic factors for patients with RCC.

Moreover, we found that RCC patients with decreased TRIM21 expression exhibited increased SREBF1 expression (Fig. 7D-E), and SREBF1 was highly expressed in RCC tissues with low TRIM21 expression (Fig. 7F-G). These data suggest that the expression of SREBF1 may be negatively correlated with TRIM21 expression in cancer tissues, which was verified by the Pearson correlation analysis (Fig. 7H). In addition, our Kaplan–Meier analysis data revealed that patients with low TRIM21 and high SREBF1 levels exhibited the poorest OS (Fig. 7I). Overall, these clinical data indicated that TRIM21 and SREBF1 expression is negatively correlated in patients with RCC, and combined detection of these two molecules may have reliable prognostic value in RCC.

## Discussion

Abnormal glucose and lipid metabolism are the main metabolic characteristics of RCC. Compared to normal renal tissue, RCC tissues have a significantly abundant lipid profile, and enhanced lipid anabolism significantly promotes tumor progression [43]. However, the role of TRIM21 in lipid metabolism in RCC has not been reported. Fully analyzing the abnormal metabolism of RCC has important guiding significance for targeting metabolic processes to inhibit RCC progression. Here, we found that TRIM21 inhibits lipid accumulation in RCC cells and prevents renal cancer tumorigenesis. TRIM21 is a novel E3 ligase of SREBF1, and the SPRY domain of TRIM21 can bind to SREBF1 and mediate its ubiquitination degradation in a K63-linked manner. The degradation of SREBF1 leads to the decreased transcriptional expression of downstream lipid metabolism-related genes and therefore fine-tunes the lipid metabolism process of RCC.

Metabolism is a complex process that includes many enzymes and provides energy for cellular function and proliferation. A variety of metabolic enzymes involved in glycolysis, amino acid metabolism, and lipid metabolism are ubiquitination substrates for TRIM21. Under glucose deprivation, the interaction between TRIM21 and FASN was enhanced, which led to the degradation of FANS [44], and this process could be repressed by GNPAT, and it should be noted that GNPAT can also be degraded by ubiquitination and degradation through K27, K33, and K48-ubiquitin at K113, K146, and K312 [45]. SREBF1 is the core upstream molecule of lipogenesis. In this study, we found that the SPRY domain of TRIM21 can bind to SREBF1 and mediate its ubiquitination degradation, which leads to decreased lipogenesis. These results suggest that TRIM21 functions at multiple steps to attenuate lipogenesis and inhibit tumor progression, whether there are other TRIM21 substrates involved in metabolic related-pathways are needed more studies to explore.

RCC is sensitive to immune checkpoint therapy, and elevated tumor glycolysis and lactate accumulation attenuate antitumor immunity in RCC. Targeting cellular metabolism has great potential to improve responsiveness to immunotherapies [46]. Furthermore, TRIM21 was reported to be highly expressed in immune cells, such as B cells, CD4 + T cells, macrophages, neutrophils, and dendritic cells, through the Tumor Immune Estimation Resource (TIMER) database [47]. Whether TRIM21 is involved in the metabolic phenotype changes of immune cells and the crosstalk between metabolic activities mediated by TRIM21 still needs to be explored. Our previous studies found that TRIM21 inhibits RCC cell glycolysis through the ubiquitination-mediated degradation of HIF-1α. Moreover, the HIF-1/2a pathway can also promote PD-L1 expression by binding to a hypoxia-response element in the PD-L1 proximal promoter in RCC [48]. These results suggested that TRIM21 may enhance the efficiency of immune checkpoint therapy by limiting glycolysis and PD-L1 expression in RCC.

Moreover, many TRIM21substrates involved in progression and treatments have been identified in various cancers [5]. TRIM21-targeted agonists and inhibitors have great potential in cancer treatment. Several clinical trials targeting the TRIM family such as TRIM18-targeted treatment in patients with hormone-refractory metastatic prostate cancer (ClinicalTrials.gov Identifier: NCT00255606) and TRIM19-targeted treatment in patients with newly diagnosed acute promyelocytic leukemia (ClinicalTrials.gov Identifier: NCT03624270) have been recruited or completed. Only one TRIM21-targeted clinical trial has focused on its prognostic value in connective tissue diseases (ClinicalTrials.gov Identifier: NCT03565601). The Development of agonists or inhibitors targeting TRIM21 has huge potential for cancer therapy.

In RCC patient samples, we found that the expression of SREBF1 and TRIM21 was negatively correlated, and patients with low TRIM21 and high SREBF1 levels exhibited poor OS. These results indicate that TRIM21-SREBF1 may be an accurate combination indicator for the prognosis and that TRIM21-SREBF1 is a potential therapeutic target. More attention should be paid to clarifying the decreased expression mechanism of TRIM21 in RCC tissues, and other possible SREBF1 regulatory mechanisms, which may provide a better understanding of the theoretical basis for targeting TRIM21-SREBF1 molecule as a therapy for RCC.

## Conclusions

TRIM21 plays a critical role in regulating RCC lipid metabolism via decreasing SREBF1 stability, providing new insights into the function of TRIM21 and suggesting TRIM21 as a promising therapeutic target for RCC patients with RCC.

## Supplementary Information

**Additional file 1: Supplementary Figure 1.** TRIM21 decreases the expression of lipogenic enzymes by mediating ubiquitination degradation of SREBF1. TRIM21 knockdown (**A**) and overexpression in 786-O (**B**) can increase and decrease metabolic enzymes above the transcriptional level except for ACLY and SREBF. TRIM21 overexpression (**C**) and knockdown in 786-O (**D**) can decrease and increase metabolic enzymes above the protein level except for ACLY. **E** TRIM21 was overexpressed in 786-O, and immunoprecipitation was performed using an Anti-SREBF1 antibody, the results suggested that TRIM21 could bind to SREBF1 and increase the ubiquitination degradation level of SREBF1. All the results were confirmed by three times repeated experiments. All statistical tests were two-sided. *p < 0.05, **p < 0.01, ***p < 0.001, ns, no significance. **Supplementary Figure 2.** SREBF1 is critical for TRIM21-mediated lipogenesis inhibition in vitro. **A** and **B** Western blot was used to detect the transient knockdown transfection efficiency of TRIM21 and SREBF1 alone or combination with in ACHN and 786-O, and GAPDH was used as a loading control. Flow cytometry (**C**-**D**) and a microplate reader (**E**-**F**) were used to access the fluorescence intensity of Nile red staining when TRIM21 and SREBF1 were knocked down alone or simultaneously. **G** Western blot was used to detect the transfection efficiency when TRIM21 and SREBF1 were transient over-expressed alone or in combination with in 786-O, and GAPDH was used as a loading control. **H** Representative images of Oil Red O staining when TRIM21 and SREBF1 were transient over-expressed alone or in combination with 786-O cells (red arrows indicate lipid droplets). Flow cytometry (**I**) and a microplate reader (**J**) were used to access the fluorescence intensity of Nile red staining. All the results are confirmed by three times repeated experiments. Data are presented as the means ± SEM for experiments in triplicate. ns, no significance, **p < 0.01, ***p < 0.001. **Supplementary Figure 3.** The expression of TRIM21 is negatively correlated with the expression of lipogenic enzymes in RCC patients. Representative immunohistochemistry images of TRIM21, SREBF1, FASN, MGLL, and HSL protein expression in adjacent tumor tissues and carcinoma tissues in RCC patients. 

**Additional file 2: Supplementary Table 1.** TRIM21 staining and clinicopathological characteristics of 239 renal cancer patients.

**Additional file 3: Supplementary Table 2.** SREBF1 staining and clinicopathological characteristics of 239 renal cancer patients.

**Additional file 4: Supplementary Table 3.** Univariate Cox proportional regression analysis on 5-year overall survival of 239 renal cancer patients.

**Additional file 5: Supplementary Table 4. **Multivariate Cox regression analysis on 5-year overall survival of 239 renal cancer patients. 

**Additional file 6: Supplementary Table 5.** Multivariate Cox regression analysis on 5-year overall survival of 239 renal cancer patients.

**Additional file 7.**

## Data Availability

The data supporting the findings of this study are available from the corresponding author upon reasonable request.

## Abbreviations

RCC: Renal cell carcinoma
TRIM: Tripartite motif-containing protein family
FA: Fatty acid
FASN: Fatty acid synthase
GNPAT: Glyceronephosphate O-acyltransferase
SREBF1: Sterol regulatory element binding transcription factor 1
TMA: Tissue microarray
IHC: Immunohistochemistry
OCAR: Oxygen consumption rat
CPT1a: Carnitine palmitoyl transferase 1 A
LDs: Lipid droplets
CCRCC: Clear cell renal cell carcinoma
PC: Phosphatidylcholines
PE: Phosphatidyl ethanolamine
TG: Triacylglycerols
DG: Diacylglycerols
ChE: Cholesterol
ACLY: ATP citrate lyase
SCD1: Stearoyl-CoA desaturase 1
GPAM: Glycerol-3-phosphate acyltransferase mitochondrial
ATGL: Patatin-like phospholipase domain containing 2
MGLL: Monoglyceride lipase
HSL: Hormone sensitive lipase
CERS6: Ceramide synthase 6
CHX: Cycloheximide
TCGA: The Cancer Genome Atlas
OS: Overall survival
TIMER: Tumor Immune Estimation Resource database
