# Supplementary figures and images for: TRIM21 attenuates renal carcinoma lipogenesis and malignancy by regulating SREBF1 protein stability
Source: J Exp Clin Cancer Res. 2023 Jan 25;42:34. doi: 10.1186/s13046-022-02583-z (PMC9875457; doi:10.1186/s13046-022-02583-z)

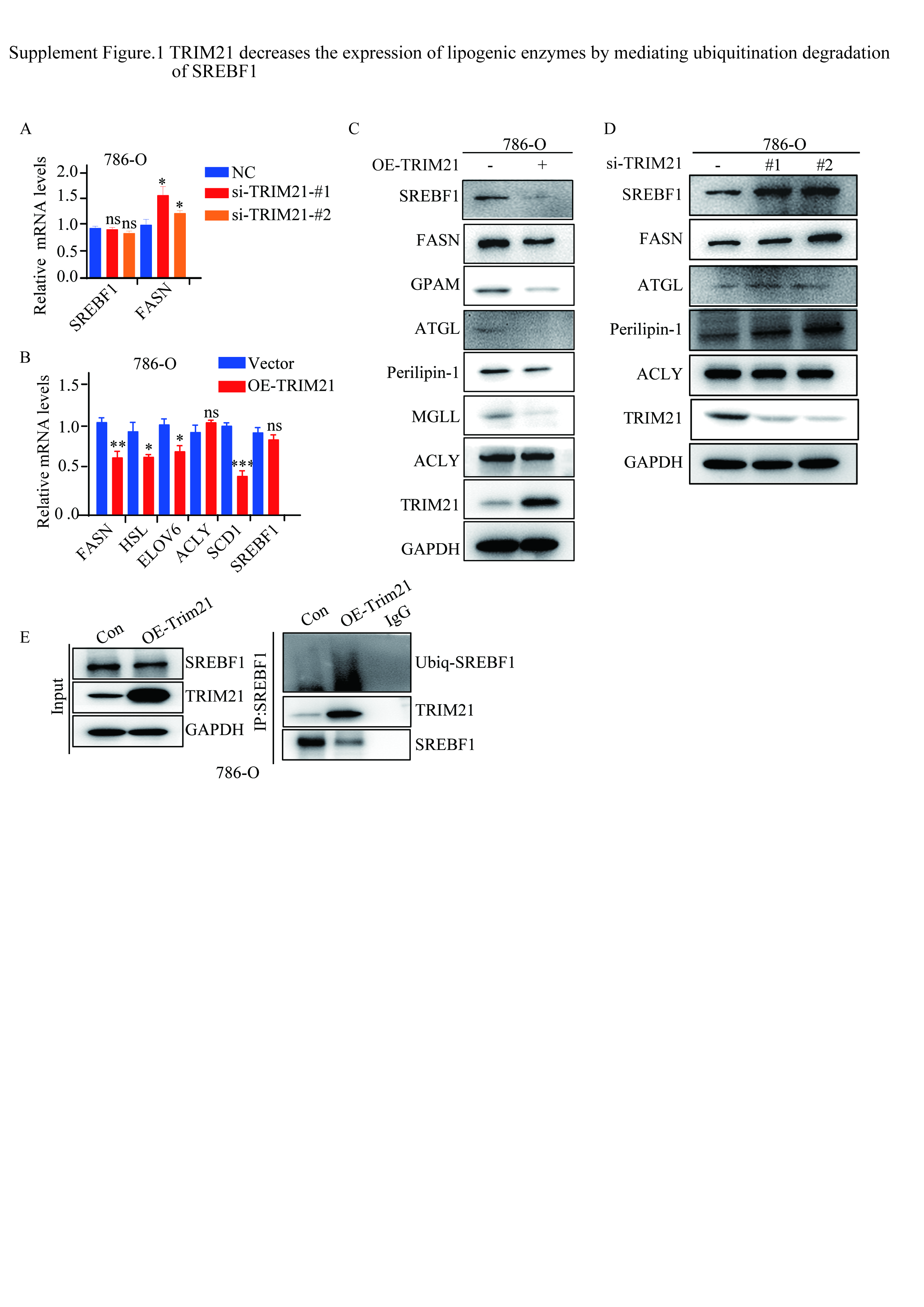

Supplement: Supplementary file 1 — Additional file 1: Supplementary Figure 1. TRIM21 decreases the expression of lipogenic enzymes by mediating ubiquitination degradation of SREBF1. TRIM21 knockdown (A) and overexpression in 786-O (B) can increase and decrease metabolic enzymes above the transcriptional level except for ACLY and SREBF. TRIM21 overexpression (C) and knockdown in 786-O (D) can decrease and increase metabolic enzymes above the protein level except for ACLY. E TRIM21 was overexpressed in 786-O, and immunoprecipitation was performed using an Anti-SREBF1 antibody, the results suggested that TRIM21 could bind to SREBF1 and increase the ubiquitination degradation level of SREBF1. All the results were confirmed by three times repeated experiments. All statistical tests were two-sided. *p < 0.05, **p < 0.01, ***p < 0.001, ns, no significance. Supplementary Figure 2. SREBF1 is critical for TRIM21-mediated lipogenesis inhibition in vitro. A and B Western blot was used to detect the transient knockdown transfection efficiency of TRIM21 and SREBF1 alone or combination with in ACHN and 786-O, and GAPDH was used as a loading control. Flow cytometry (C-D) and a microplate reader (E-F) were used to access the fluorescence intensity of Nile red staining when TRIM21 and SREBF1 were knocked down alone or simultaneously. G Western blot was used to detect the transfection efficiency when TRIM21 and SREBF1 were transient over-expressed alone or in combination with in 786-O, and GAPDH was used as a loading control. H Representative images of Oil Red O staining when TRIM21 and SREBF1 were transient over-expressed alone or in combination with 786-O cells (red arrows indicate lipid droplets). Flow cytometry (I) and a microplate reader (J) were used to access the fluorescence intensity of Nile red staining. All the results are confirmed by three times repeated experiments. Data are presented as the means ± SEM for experiments in triplicate. ns, no significance, **p < 0.01, ***p < 0.001. Supplementary Figure 3. T [file 13046_2022_2583_MOESM1_ESM.zip › Supplement Figure.1.jpg]

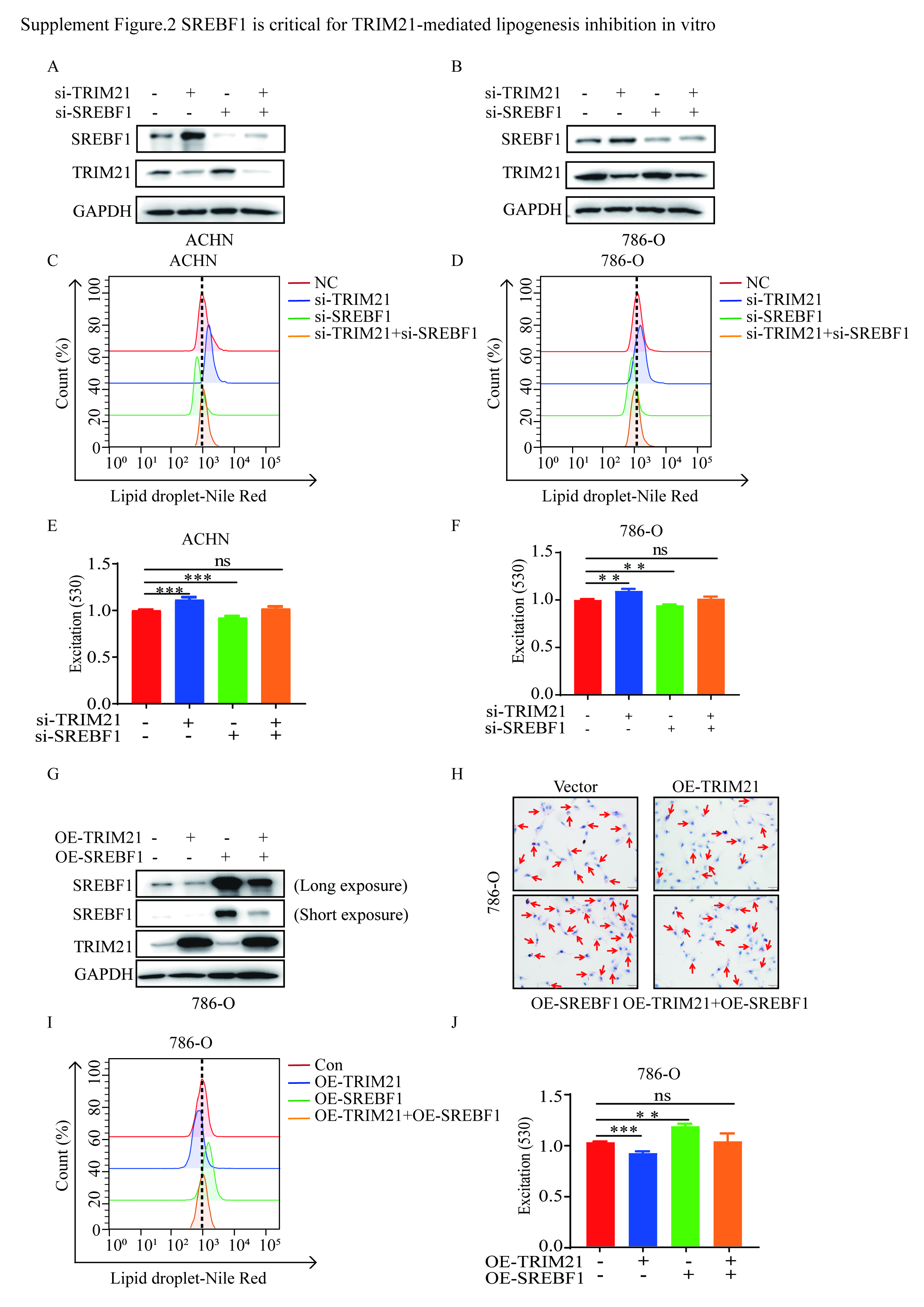

Supplement: Supplementary file 1 — Additional file 1: Supplementary Figure 1. TRIM21 decreases the expression of lipogenic enzymes by mediating ubiquitination degradation of SREBF1. TRIM21 knockdown (A) and overexpression in 786-O (B) can increase and decrease metabolic enzymes above the transcriptional level except for ACLY and SREBF. TRIM21 overexpression (C) and knockdown in 786-O (D) can decrease and increase metabolic enzymes above the protein level except for ACLY. E TRIM21 was overexpressed in 786-O, and immunoprecipitation was performed using an Anti-SREBF1 antibody, the results suggested that TRIM21 could bind to SREBF1 and increase the ubiquitination degradation level of SREBF1. All the results were confirmed by three times repeated experiments. All statistical tests were two-sided. *p < 0.05, **p < 0.01, ***p < 0.001, ns, no significance. Supplementary Figure 2. SREBF1 is critical for TRIM21-mediated lipogenesis inhibition in vitro. A and B Western blot was used to detect the transient knockdown transfection efficiency of TRIM21 and SREBF1 alone or combination with in ACHN and 786-O, and GAPDH was used as a loading control. Flow cytometry (C-D) and a microplate reader (E-F) were used to access the fluorescence intensity of Nile red staining when TRIM21 and SREBF1 were knocked down alone or simultaneously. G Western blot was used to detect the transfection efficiency when TRIM21 and SREBF1 were transient over-expressed alone or in combination with in 786-O, and GAPDH was used as a loading control. H Representative images of Oil Red O staining when TRIM21 and SREBF1 were transient over-expressed alone or in combination with 786-O cells (red arrows indicate lipid droplets). Flow cytometry (I) and a microplate reader (J) were used to access the fluorescence intensity of Nile red staining. All the results are confirmed by three times repeated experiments. Data are presented as the means ± SEM for experiments in triplicate. ns, no significance, **p < 0.01, ***p < 0.001. Supplementary Figure 3. T [file 13046_2022_2583_MOESM1_ESM.zip › Supplement Figure.2.jpg]

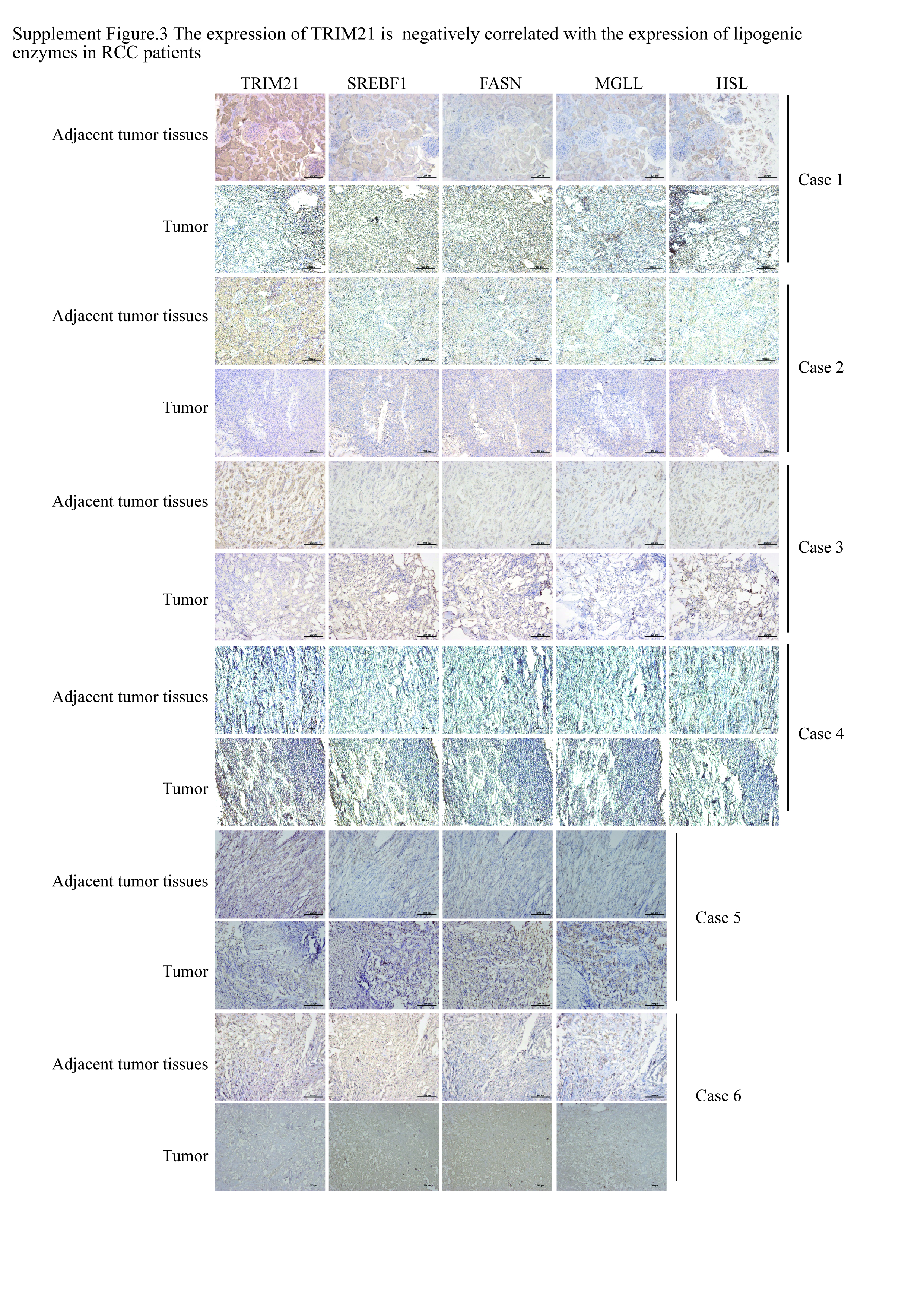

Supplement: Supplementary file 1 — Additional file 1: Supplementary Figure 1. TRIM21 decreases the expression of lipogenic enzymes by mediating ubiquitination degradation of SREBF1. TRIM21 knockdown (A) and overexpression in 786-O (B) can increase and decrease metabolic enzymes above the transcriptional level except for ACLY and SREBF. TRIM21 overexpression (C) and knockdown in 786-O (D) can decrease and increase metabolic enzymes above the protein level except for ACLY. E TRIM21 was overexpressed in 786-O, and immunoprecipitation was performed using an Anti-SREBF1 antibody, the results suggested that TRIM21 could bind to SREBF1 and increase the ubiquitination degradation level of SREBF1. All the results were confirmed by three times repeated experiments. All statistical tests were two-sided. *p < 0.05, **p < 0.01, ***p < 0.001, ns, no significance. Supplementary Figure 2. SREBF1 is critical for TRIM21-mediated lipogenesis inhibition in vitro. A and B Western blot was used to detect the transient knockdown transfection efficiency of TRIM21 and SREBF1 alone or combination with in ACHN and 786-O, and GAPDH was used as a loading control. Flow cytometry (C-D) and a microplate reader (E-F) were used to access the fluorescence intensity of Nile red staining when TRIM21 and SREBF1 were knocked down alone or simultaneously. G Western blot was used to detect the transfection efficiency when TRIM21 and SREBF1 were transient over-expressed alone or in combination with in 786-O, and GAPDH was used as a loading control. H Representative images of Oil Red O staining when TRIM21 and SREBF1 were transient over-expressed alone or in combination with 786-O cells (red arrows indicate lipid droplets). Flow cytometry (I) and a microplate reader (J) were used to access the fluorescence intensity of Nile red staining. All the results are confirmed by three times repeated experiments. Data are presented as the means ± SEM for experiments in triplicate. ns, no significance, **p < 0.01, ***p < 0.001. Supplementary Figure 3. T [file 13046_2022_2583_MOESM1_ESM.zip › Supplement Figure.3.jpg]
