# Supplementary material for: TRIM21 attenuates renal carcinoma lipogenesis and malignancy by regulating SREBF1 protein stability
Source: J Exp Clin Cancer Res. 2023 Jan 25;42:34. doi: 10.1186/s13046-022-02583-z (PMC9875457; doi:10.1186/s13046-022-02583-z)
Supplement: Supplementary file 2 — Additional file 2: Supplementary Table 1. TRIM21 staining and clinicopathological characteristics of 239 renal cancer patients. [file 13046_2022_2583_MOESM2_ESM.doc]

**Supplementary Table 1** TRIM21 staining and clinicopathological characteristics of 239 renal cancer patients.

| **Variables** | **TRIM21 staining** | | | |
| --- | --- | --- | --- | --- |
|  | **Low (%)** | **High (%)** | **Total** | ***P* *** |
| **Age** |  |  |  |  |
| ≤56 | 71(60.7) | 46(39.3) | 117 | 0.438 |
| >56 | 68(55.7) | 54(44.3) | 122 |  |
| **Gender** |  |  |  |  |
| Male | 85(55.2) | 69(44.8) | 154 | 0.211 |
| Female | 54(63.5) | 31(36.5) | 85 |  |
| **Tumor size** |  |  |  |  |
| T1(≤7cm) | 76(50.3) | 75(49.7) | 151 | 0.001 |
| T2(>7cm) | 63(71.6) | 25(28.4) | 88 |  |
| **Depth of invasion** |  |  |  |  |
| Intrarenal | 89(57.4) | 66(42.6) | 155 | 0.753 |
| Extrarenal | 50(59.5) | 34(40.5) | 84 |  |
| **Lymph node metastasis** |  |  |  |  |
| Negative | 60(49.2) | 62(50.8) | 122 | 0.004 |
| Positive | 79(67.5) | 38(32.5) | 117 |  |
| **Distant metastasis** |  |  |  |  |
| Negative | 65(48.5) | 69(51.5) | 134 | 0.001 |
| Positive | 74(70.5) | 31(29.5) | 105 |  |
| **Urinary system diseases** |  |  |  |  |
| Negative | 126(56.5) | 97(43.5) | 223 | 0.053 |
| Positive | 13(81.2) | 3(18.8) | 16 |  |

^*^ *P* values are from χ^2^ test.
