# Supplementary material for: TRIM21 attenuates renal carcinoma lipogenesis and malignancy by regulating SREBF1 protein stability
Source: J Exp Clin Cancer Res. 2023 Jan 25;42:34. doi: 10.1186/s13046-022-02583-z (PMC9875457; doi:10.1186/s13046-022-02583-z)
Supplement: Supplementary file 3 — Additional file 3: Supplementary Table 2. SREBF1 staining and clinicopathological characteristics of 239 renal cancer patients. [file 13046_2022_2583_MOESM3_ESM.doc]

**Supplementary Table 2 SREBF1 staining and clinicopathological characteristics of 239 renal cancer patients.**

| **Variables** | **SREBF1 staining** | | | | |
| --- | --- | --- | --- | --- | --- |
|  | **Low (%)** | **High (%)** | **Total** | | ***P* *** |
| **Age** |  |  |  |  | |
| ≤56 | 48(41.0) | 69(59.0) | 117 | 0.254 | |
| >56 | 59(48.4) | 63(51.6) | 122 |  | |
| **Gender** |  |  |  |  | |
| Male | 63(40.9) | 91(59.1) | 154 | 0.106 | |
| Female | 44(51.8) | 41(48.2) | 85 |  | |
| **Tumor size** |  |  |  |  | |
| T1(≤7cm) | 78(51.7) | 73(48.3) | 151 | 0.005 | |
| T2(>7cm) | 29(33.0) | 59(67.0) | 88 |  | |
| **Depth of invasion** |  |  |  |  | |
| Intrarenal | 70(45.2) | 85(54.8) | 155 | 0.869 | |
| Extrarenal | 37(44.0) | 47(56.0) | 84 |  | |
| **Lymph node metastasis** |  |  |  |  | |
| Negative | 66(54.1) | 56(45.9) | 122 | 0.003 | |
| Positive | 41(35.0) | 76(65.0) | 117 |  | |
| **Distant metastasis** |  |  |  |  | |
| Negative | 77(57.5) | 57(42.5) | 134 | 0.000 | |
| Positive | 30(28.6) | 75(71.4) | 105 |  | |
| **Urinary system diseases** |  |  |  |  | |
| Negative | 99(44.4) | 124(55.6) | 223 | 0.663 | |
| Positive | 8(50.0) | 8(50.0.8) | 16 |  | |

^*^ *P* values are from χ^2^ test.
