# Supplementary material for: TRIM21 attenuates renal carcinoma lipogenesis and malignancy by regulating SREBF1 protein stability
Source: J Exp Clin Cancer Res. 2023 Jan 25;42:34. doi: 10.1186/s13046-022-02583-z (PMC9875457; doi:10.1186/s13046-022-02583-z)
Supplement: Supplementary file 4 — Additional file 4: Supplementary Table 3. Univariate Cox proportional regression analysis on 5-year overall survival of 239 renal cancer patients. [file 13046_2022_2583_MOESM4_ESM.doc]

**Supplementary Table 3** Univariate Cox proportional regression analysis on 5-year overall survival of 239 renal cancer patients.

| Variable^*^ | Overall survival | | |
| --- | --- | --- | --- |
|  | Hazard ratio | 95% CI^†^ | *P*^*^ |
| **TRIM21** |  |  |  |
| Low | 1.000 |  |  |
| High | 0.426 | 0.303-0.600 | 0.000 |
| **SREBF1** |  |  |  |
| Low | 1.000 |  |  |
| High | 3.721 | 2.626-5.274 | 0.000 |
| **Age** |  |  |  |
| ≤56 years | 1.000 |  |  |
| >56 years | 0.886 | 0.647-1.213 | 0.45 |
| **Gender** |  |  |  |
| Male | 1.000 |  |  |
| Female | 1.003 | 0.722-1.393 | 0.988 |
| **Tumor size** |  |  |  |
| ≤7 cm | 1.000 |  |  |
| >7 cm | 1.987 | 1.446-2.731 | 0.000 |
| **Depth of invasion** |  |  |  |
| Intra-renal | 1.000 |  |  |
| Extra-renal | 1.814 | 1.321-2.491 | 0.000 |
| **Lymph node metastasis** |  |  |  |
| Negative | 1.000 |  |  |
| positive | 1.735 | 1.261-2.386 | 0.001 |
| **Distant metastasis** |  |  |  |
| Negative | 1.000 |  |  |
| positive | 3.444 | 2.485-4.775 | 0.000 |
| **Urinary system diseases** |  |  |  |
| Negative | 1.000 |  |  |
| positive | 0.743 | 0.364-1.517 | 0.415 |

* *P* values are from Log-rank test. † CI: confidence interval.
