# Supplementary material for: TRIM21 attenuates renal carcinoma lipogenesis and malignancy by regulating SREBF1 protein stability
Source: J Exp Clin Cancer Res. 2023 Jan 25;42:34. doi: 10.1186/s13046-022-02583-z (PMC9875457; doi:10.1186/s13046-022-02583-z)
Supplement: Supplementary file 5 — Additional file 5: Supplementary Table 4. Multivariate Cox regression analysis on 5-year overall survival of 239 renal cancer patients. [file 13046_2022_2583_MOESM5_ESM.doc]

**Supplementary Table 4** Multivariate Cox regression analysis on 5-year overall survival of 239 renal cancer patients.

| Variable^*^ | Overall survival | | | |  |
| --- | --- | --- | --- | --- | --- |
|  | Hazard ratio | | 95% CI^†^ | *P* |  |
| TRIM21 | 0.604 | 0.419 to 0.87 | | 0.007 |  |
| Gender | 0.938 | 0.67 to 1.311 | | 0.706 |  |
| Tumor size | 1.667 | 1.201 to 2.313 | | 0.002 |  |
| Depth of invasion | 1.608 | 1.16 to 2.228 | | 0.004 |  |
| Lymph node metastasis | 1.438 | 1.038 to 1.992 | | 0.029 |  |
| Distant metastasis | 2.881 | 2.062 to 4.052 | | 0.000 |  |

^*^Coding of variables: TRIM21 was coded as 1 (low), and 2 (high). Gender was coded as 1 (male), and 2 (female). Tumor size was coded as 1 (≤7 cm), and 2 (>7 cm). Depth of invasion was coded as 1 (intra-renal), and 2 (extra-renal). Lymph node metastasis was coded as 1 (negative), and 2 (positive). Distance metastasis was coded as 1 (negative), and 2 (positive).

^†^ CI: confidence interval.
