# Supplementary material for: TRIM21 attenuates renal carcinoma lipogenesis and malignancy by regulating SREBF1 protein stability
Source: J Exp Clin Cancer Res. 2023 Jan 25;42:34. doi: 10.1186/s13046-022-02583-z (PMC9875457; doi:10.1186/s13046-022-02583-z)
Supplement: Supplementary file 6 — Additional file 6: Supplementary Table 5. Multivariate Cox regression analysis on 5-year overall survival of 239 renal cancer patients. [file 13046_2022_2583_MOESM6_ESM.doc]

**Supplementary Table 5** Multivariate Cox regression analysis on 5-year overall survival of 239 renal cancer patients.

| Variable^*^ | Overall survival | | | |  |
| --- | --- | --- | --- | --- | --- |
|  | Hazard ratio | | 95% CI^†^ | *P* |  |
| SREBF1 | 3.17 | 2.200 to 4.568 | | 0.000 |  |
| Gender | 1.092 | 0.783 to 1.522 | | 0.605 |  |
| Tumor size | 1.876 | 1.348 to 2.612 | | 0.000 |  |
| Depth of invasion | 1.876 | 1.348 to 2.612 | | 0.000 |  |
| Lymph node metastasis | 1.226 | 0.876 to 1.715 | | 0.234 |  |
| Distant metastasis | 2.645 | 1.891 to 3.698 | | 0.000 |  |

^*^Coding of variables: SREBF1 was coded as 1 (low), and 2 (high). Gender was coded as 1 (male), and 2 (female). Tumor size was coded as 1 (≤7 cm), and 2 (>7 cm). Depth of invasion was coded as 1 (intra-renal), and 2 (extra-renal). Lymph node metastasis was coded as 1 (negative), and 2 (positive). Distance metastasis was coded as 1 (negative), and 2 (positive).

^†^ CI: confidence interval.
