# Supplementary material for: TRIM21 attenuates renal carcinoma lipogenesis and malignancy by regulating SREBF1 protein stability
Source: J Exp Clin Cancer Res. 2023 Jan 25;42:34. doi: 10.1186/s13046-022-02583-z (PMC9875457; doi:10.1186/s13046-022-02583-z)
Supplement: Supplementary file 7 — Additional file 7. [file 13046_2022_2583_MOESM7_ESM.doc]

**TRIM21 attenuates renal carcinoma lipogenesis and malignancy by regulating SREBF1 protein stability**

Xintian Chen^#1,2,3^, Hongmei Yong^#4^, Miaolei Chen^#1^, Chuying Deng^1^, Pengfei Wang^1^, Sufang Chu^1^, Minle Li^1,2,3^, Pingfu Hou^1,2,3^, Junnian Zheng*^1,2,3^, Zhongwei Li*^1,2,3^ and Jin Bai*^1,2,3^

^1^Cancer Institute, Xuzhou Medical University, Xuzhou, Jiangsu, China.

^2^Center of Clinical Oncology, the Affiliated Hospital of Xuzhou Medical University, Xuzhou, Jiangsu, China.

^3^Jiangsu Center for the Collaboration and Innovation of Cancer Biotherapy, Cancer Institute, Xuzhou Medical University, Xuzhou, Jiangsu, China.

^4^Department of Oncology, The Affiliated Huai'an Hospital of Xuzhou Medical University and The Second People's Hospital of Huai'an, Huaian, Jiangsu, China

**Authorship notes:** #These authors contributed equally to this work.

**Running title**: TRIM21 inhibits lipid metabolism through SREBF1.

***Correspondence Authors:**

Jin Bai, Cancer Institute, Xuzhou Medical University, 209 Tongshan Road, Xuzhou, 221004, Jiangsu Province, China. E-mail: bj@xzhmu.edu.cn.

Zhongwei Li, Cancer Institute, Xuzhou Medical University, 209 Tongshan Road, Xuzhou, 221004,Jiangsu Province, China. E-mail: lizw074@xzhmu.edu.cn.

Junnian Zheng, Cancer Institute, Xuzhou Medical University. 209 Tongshan Road, Xuzhou, 221004, Jiangsu Province, China. E-mail: jnzheng@xzhmu.edu.cn.

**Conflict of interest:** No potential conflicts of interest were disclosed by the authors.

**Supplementary Materials**

**Supplementary Materials Table.1**

**The sequences of siRNAs used for gene silencing**

|  | Sense | Anti-sense |
| --- | --- | --- |
| si-TRIM21-NC | UUCUCCGAACGUGUCACGUTT | ACGUGACACGUUCGGAGAATT |
| si-TRIM21-#1 | GACUUCACCUGUUCUGUGATT | UCACAGAACAGGUGAAGUCTT |
| si-TRIM21-#2 | CAGCACGCUUGACAAUGAUTT | AUCAUUGUCAAGCGUGCUGTT |
| si-SREBF1-NC | UUCUCCGAACGUGUCACGUTT | ACGUGACACGUUCGGAGAATT |
| si-SREBF1-#1 | CCUAUUUGACCCACCCUAUTT | AUAGGGUGGGUCAAAUAGGTT |
| si-SREBF1-#2 | CCAUCGACUACAUUCGCUUTT | AAGCGAAUGUAGUCGAUGGTT |

**Supplementary Materials Table.2**

**The primers used for quantitative RT-PCR**

| **Primer Name** | **Sequence 5’-3’** |
| --- | --- |
| GAPDH Forward | AAGGTCGGAGTCAACGGATTTG |
| GAPDH Reverse | CCATGGGTGGAATCATATTGGAA |
| FASN Forward | AAGGACCTGTCTAGGTTTGATGC |
| FASN Reverse | TGGCTTCATAGGTGACTTCCA |
| GPAM Forward | GATGTAAGCACACAAGTGAGGA |
| GPAM Reverse | TCCGACTCATTAGGCTTTCTTTC |
| MGLL Forward | ATGCCAGAGGAAAGTTCCCC |
| MGLL Reverse: | CGTCTGCATTGACCAGGTG |
| HSL Forward | TGAGGGAATGCGATAAGTTGC |
| HSL Reverse | TGGTCCAGTTCGTCTGAGTATT |
| Perilipin-1 Forward | AAGGCCCTGAAGTGGGTTC |
| Perilipin-1 Reverse | GCATGTGGTCTATCAGCTCCA |
| ELOV6 Forward | AACGAGCAAAGTTTGAACTGAGG |
| ELOV6 Reverse | TCGAAGAGCACCGAATATACTGA |
| CERBS6 Forward | GCAGGGATCTTAGCCTGGTTC |
| CERBS6 Reverse | AAAAGCGAGATAGAGGTCCTCA |
| LIPIN-1 Forward | CGGAGGGTCAGGTCGTTTC |
| LIPIN-1 Reverse | CAACTTCTCCATGCGGAACTC |
| ACLY Forward | TCGGCCAAGGCAATTTCAGAG |
| ACLY Reverse | CGAGCATACTTGAACCGATTCT |
| LIPIN-1 Forward | CGGAGGGTCAGGTCGTTTC |
| LIPIN-1 Reverse | CAACTTCTCCATGCGGAACTC |
| SCD Forward | GAGGCACCTACATTGGATGCT |
| SCD Reverse | CGTAGACATAGGACCGCTCA |
| SREBF1 Forward | GCCCCTGTAACGACCACTG |
| SREBF1 Reverse | CAGCGAGTCTGCCTTGATG |
| ACC Forward | AGTCATCGGTCAGACACCCTT |
| ACC Reverse | GTGCAGCGTTATCTCCAACAG |

**Supplementary Methods**

**1. RNA extract, reverse transcription-PCR, and qRT-PCR**

RNA was extracted using TRIzol (Invitrogen) and cDNA was synthesized using the HiScript 1^st^ Strand cDNA Synthesis Kit (Vazyme Biotech, Nanjing, China). Real-time PCR analysis was carried out on ABI-7500 using UltraSYBR One-Step RT-qPCR Kit (CWBIO, Beijing, China).

**2. Assessment of IHC**

Three pathologists assessed separately the TMAs under blinded experimental conditions and all differences that arise were resolved by discussion. The staining scores of TRIM21 and SREBF1 were evaluated by combining the percentage of cells with the staining intensity and being dependent on the IRS (immunoreactivity score, IRS). The intensity of TRIM21 and SREBF1 immunostaining was scored as 0–3 (0, negative; 1, weak; 2, moderate; 3, strong); the percentage of immunoreactivity cells was graded as 1 (0–25%), 2 (26–50%), 3 (51–75%), and 4 (76–100%). Relied on the IRS, the level of TRIM21 expression was categorized as low (IRS: 0–2) and high (IRS: 3–12) expression, and the level of SREBF1 expression was categorized as low (IRS: 0–4) and high (IRS: 6–12) expression.

**3. Seahorse assays**

In brief, after the knockdown of TRIM21 for 24h, cells were plated in the wells of 96-well plates (1×10^4^ cells/well; XF96 plates; Seahorse Bioscience) and incubated overnight at 37°C. The next day, the medium was changed to XF assay medium and loaded with medium or etomoxir, oligomycin, FCCP, and rotenone/antimycin A respectively, according to the manufacturer’s recommendation. All the tests were performed on the XFe96 bioanalyzer.

**4. Oil Red O staining**

Briefly, the working solution was proportioned as described in the manufacturer’s instructions. The cells were washed with PBS twice and then fixed with 4% paraformaldehyde at room temperature for 10min. After washing with PBS, soak and wash with 60% isopropyl alcohol. Dye with Oil Red O working solution for 10min; 60% isopropanol differentiation to clear interstitial; Hematoxylin (22190; Fanbo Biochemicals Co., Ltd., Beijing, China) was counterstained for 1min after washing with PBS. PBS washes away excess hematoxylin, and staining was assessed by bright field microscopy.

**5. Generation of stable cells using lentivirus**

TRIM21 cDNA was cloned to the pCDH1-CMV-MSC-EF1-Puro vector. Lentiviruses were produced by co-transfecting HEK293T cells with one of the expression plasmids and the packaging plasmids (psPAX2 and pMD2.G). The supernatants were collected after 48 hours and filtered through 0.45μm filters (Millipore, Temecula, CA, USA), then concentrated using Amicon Ultra centrifugal filters (Millipore 100KD MWCO). The concentrated viruses were used to infect luciferase-labeled ACHN cells (ACHN-luc). And the stable transfection cell lines were selected with 2mg/ml puromycin. Then the stably overexpressed or knocked-down SREBF1 and TRIM21 ACHN-luc cells were further selected and identified.

**6. Database analysis relevance and clinical significance of TRIM21 and SREBF1**

Converting counts data to TPM and normalizing the data log2 (TPM+1), keeping samples with clinical information at the same time. Finally, there are 532 samples for subsequent analysis. Log-rank test was used to compare differences in survival between these groups. The timeROC (v 0.4) analysis was used to compare the predictive accuracy of SREBF1 and TRIM21, and the risk score. Multivariate cox regression analysis was used to construct a prognostic model, and the R package survival was used for the analysis. For Kaplan-Meier curves, p-values and hazard ratio (HR) with 95% confidence interval (CI) were generated by log-rank tests and univariate cox proportional hazards regression. All the analysis methods and R packages were implemented by R (foundation for statistical computing 2020) version 4.0.3. *p*<0.05 was considered statistically significant.

**Supplementary Figure legends**

**Supplementary Figure.1 TRIM21 decreases the expression of lipogenic enzymes by mediating ubiquitination degradation of SREBF1**

TRIM21 knockdown **(A)** and overexpression in 786-O **(B)** can increase and decrease

metabolic enzymes above the transcriptional level except for ACLY and SREBF. TRIM21 overexpression **(C)** and knockdown in 786-O **(D)** can decrease and increase

metabolic enzymes above the protein level except for ACLY. **(E)** TRIM21 was overexpressed in 786-O, and immunoprecipitation was performed using an Anti-SREBF1 antibody, the results suggested that TRIM21 could bind to SREBF1 and increase the ubiquitination degradation level of SREBF1. All the results were confirmed by three times repeated experiments. All statistical tests were two-sided. **p* < 0.05, ***p* < 0.01, ****p* < 0.001, ns, no significance.

**Supplementary Figure.2 SREBF1 is critical for TRIM21-mediated lipogenesis inhibition *in vitro*. (A and B)** Western blot was used to detect the transient knockdown transfection efficiency of TRIM21 and SREBF1 alone or combination with in ACHN and 786-O, and GAPDH was used as a loading control. Flow cytometry **(C-D)** and a microplate reader **(E-F)** were used to access the fluorescence intensity of Nile red staining when TRIM21 and SREBF1 were knocked down alone or simultaneously. **(G)** Western blot was used to detect the transfection efficiency when TRIM21 and SREBF1 were transient over-expressed alone or in combination with in 786-O, and GAPDH was used as a loading control. **(H)** Representative images of Oil Red O staining when TRIM21 and SREBF1 were transient over-expressed alone or in combination with 786-O cells (red arrows indicate lipid droplets). Flow cytometry **(I)** and a microplate reader **(J)** were used to access the fluorescence intensity of Nile red staining. All the results are confirmed by three times repeated experiments. Data are presented as the means ± SEM for experiments in triplicate. ns, no significance, ***p* < 0.01, ****p* < 0.001.

**Supplementary Figure.3 The expression of TRIM21 is negatively correlated with the expression of lipogenic enzymes in RCC patients.** Representative immunohistochemistry images of TRIM21, SREBF1, FASN, MGLL, and HSL protein expression in adjacent tumor tissues and carcinoma tissues in RCC patients.
